# BRCA1 interactors, RAD50 and BRIP1, as prognostic markers for triple-negative breast cancer severity

**DOI:** 10.3389/fgene.2023.1035052

**Published:** 2023-02-16

**Authors:** Muhseena N. Katheeja, Shankar Prasad Das, Ranajit Das, Suparna Laha

**Affiliations:** Yenepoya Research Centre, Yenepoya (Deemed to be) University, Mangalore, Karnataka, India

**Keywords:** data curation, TCGA, overall survival, tumor suppressor, homologous recombination, non-homologous end-joining repair, DNA damage

## Abstract

**Introduction:** BRIP1 (BRCA1-interacting protein 1) is one of the major interacting partners of BRCA1, which plays an important role in repair by homologous recombination (HR). This gene is mutated in around 4% of cases of breast cancer; however, its mechanism of action is unclear. In this study, we presented the fundamental role of BRCA1 interactors BRIP1 and RAD50 in the development of differential severity in triple-negative breast cancer (TNBC) among various affected individuals.

**Methods:** We have analyzed the expression of DNA repair-related genes in different BC cells using Real-time PCR and western blotting analysis and assessed changes in stemness property and proliferation through Immunophenotyping. We have performed cell cycle analysis to see the defect in checkpoints and also immunofluorescence assay to confirm the accumulation of gamma-H2AX and BRCA1 foci and subsequent incidence. We have performed a severity analysis using TCGA data sets for comparing the expression in MDA-MB-468 MDA-MB-231 and MCF7 cell line.

**Results:** We showed that in some TNBC cell lines such as MDA-MB-231, the functioning of both BRCA1/TP53 is compromised. Furthermore, the sensing of DNA damage is affected. Due to less damage-sensing capability and low availability of BRCA1 at the damage sites, the repair by HR becomes inefficient, leading to more damage. Accumulation of damage sends a signal for over activation of NHEJ repair pathways. Over expressed NHEJ molecules with compromised HR and checkpoint conditions lead to higher proliferation and error-prone repair, which increases the mutation rate and corresponding tumour severity. The in-silico analysis of the TCGA datasets with gene expression in the deceased population showed a significant correlation of BRCA1 expression with overall survival (OS) in TNBCs (0.0272). The association of BRCA1 with OS became stronger with the addition of BRIP1 expression (0.000876**).

**Conclusion:** The severity phenotypes were more in cells having compromised BRCA1–BRIP1 functioning. Since the OS is directly proportional to the extent of severity, the data analysis hints at the role of BRIP1 in controlling the severity of TNBC.

## Introduction

Breast cancer (BC) is the second leading cause of cancer-related death in women (11.7% of all cases) and was identified to be a heterogeneous disease at the molecular level (Sung et al., 2021). Estrogen receptor (ER), progesterone receptor (PR), and human epidermal growth factor receptor 2 (HER2) markers are used for prognostic identification of BC, followed by appropriate targeted therapies (Dai et al., 2016). For a better understanding of the heterogeneity of breast cancer, it is classified into six intrinsic subtypes, namely, luminal A, luminal B, HER2-enriched, claudin-low, basal-like, and a normal breast-like group (Dai et al., 2015). TNBC is characterized by its absence of the target receptors such as ER, PR, and HER2. An estimated 1,70,000 cases (10%–20%) of those diagnosed with breast cancer were categorized as TNBC (Aysola et al., 2013). The incidence rate of TNBC is higher in the Indian population, with a prevalence of 25.04%, than in the Western population and African–American (AA) women, with prevalence rates of 12%–17% and 23.7%, respectively (Prakash et al., 2020; Sarkar and Akhtar, 2022) Due to a lack of receptors, TNBC cases show poor prognosis, followed by aggressive behavior which leads to increased metastasis and decreased survival rate (Yadav et al., 2015). Specific target molecules and their interactors and mechanism of action at the molecular level remain unclear in TNBC. The BC susceptibility genes involved in breast cancer which cause moderate risk are *BRIP1, CHEK2*, *ATM*, *RAD51C*, and *PALB2* (Foretova et al., 2019)*.* The mutations in these genes have a high risk of developing breast cancer. Therefore, an increased expression of all these genes is associated with a worse prognosis (Li et al., 2018; Konde et al., 2020; Stolarova et al., 2020; Khan and Khan, 2021). Several studies have investigated the role played by *TP53* and *BRCA1* genes and their mutations. TP53 has a role in activating the cell cycle checkpoints. Breast cancers with *BRCA1* mutation carriers are frequently categorized as triple-negative basal-like, and their prevalence rate is around 80%–90% (Godet and Gilkes, 2017). But the difference in the pathological conditions within TNBC cases suggests that only mutation in BRCA1 may or may not affect its functioning; the condition develops depending on the position of the mutation. *TP53* mutation is found in 20%–35% of breast tumors, and the mutation rate increase in TNBC to >90% (Chae et al., 2009; Chen, 2016). Recent clinical studies suggest that *BRCA1* ^*+*^ *TP53* ^+^ TNBC cases had better OS chances than *BRCA1* ^−^ *TP53* ^−^ cases and, in fact, the survival ability of breast cancer patients goes down when both *BRCA1* and *TP53* are affected (Kim et al., 2016). The aggressiveness in the disease manifestation within the TNBC group shows individual variation. Lack of receptors and malfunctioning of *TP53/BRCA1* are unlikely to be the only reason for this. Instead, it is a condition that develops due to the interplay of several genes (Feng et al., 2018). The current study aimed at addressing the molecular association of BRCA1 and TP53 with the severity of breast cancer manifestation.

BRIP1 is a helicase that interacts with BRCA1 through the BRCT domain and contributes to the DNA damage repair function of BRCA1 through homologous recombination (Muhseena et al., 2021). Mutation at the interacting domains leads to the loss of BRCA1–BRIP1 interaction, and consequently, fewer repair molecules reach the damaged sites. So, the interaction of BRCA1 with BRIP1 ensures the suppression of the mutation-prone non-homologous end-joining mechanism of repair and promotes double-strand DNA repair *via* the activation of HR (Cantor et al., 2001). The repair process also depends on several damage-sensing molecules. MRN complex consisting of RAD50, MRE11, and NBS1 plays a major role in DNA damage detection and maintaining active ATMs at the site of double-strand DNA break (Lavin, 2007; Syed and Tainer, 2018). The active ATMs phosphorylate H2AX, which amplifies damage signals and accumulates numerous DNA damage repair (DDR) proteins such as BRIP1 at double-strand breaks (DSBs) to form damage-induced foci. Once the γ-H2AX forms the foci, other DNA damage response proteins gather and bind to the site of damage, accumulate, and activate the repair activity (Lowndes and Toh, 2005). H2AX and the associated proteins such as BRIP1 also promote holding the broken ends together, thereby giving time and favorable conditions for DNA repair and minimizing the risk of misrepair (Yuan et al., 2010).

In this paper, we present an *in silico* comparative study with triple-negative and luminal breast cancer patient samples supported by *in vitro* experiments with cell lines of their respective subgroups. The study focuses on the expression and specific functional roles played by the major breast cancer tumor suppressor genes *BRCA1* and *TP53* and the BRCA1 interactor genes *BRIP1* and *RAD50*. We also performed *in silico* analysis on the expression level of the same genes in tissues at similar disease conditions. The TNBC and luminal subtypes were compared with solid normal tissue from two different tools, namely, the UCSC Xena and cBioPortal, which analyzed their correlation and roles played in the prognosis of the disease. We observed a differential expression pattern of TP53 and BRCA1 and the integrity of nuclear arrangement within different TNBC lines. The DNA damage sensing and response mechanisms through BRIP1 also change significantly with different TNBC lines. In certain TNBC conditions, due to inefficient repair and in the absence of checkpoint activation, stemness behavior, metastatic property, and high proliferation rate develop, making the tumor more aggressive and severe. The combined expression pattern of BRCA1, BRIP1, and TP53 is highly associated with the diseased population of TNBC patients (*p*-value 0.0055**) compared to the luminal type and control. In conclusion, the overall survivability is deeply affected when overexpression of BRIP1 is combined with compromised expression of BRCA1 and TP53.

## Materials and methods

### Cell line and cell culture

Human TNBC cell lines MDA-MB-231 and MDA-MB-468 and luminal cell line MCF-7 were purchased from the National Centre for Cell Science, Pune, India. The cell line was cultured as given in the datasheet provided by the repository. All cultured cell lines were free from *mycoplasma* infections.

### Total RNA isolation and qRT-PCR for mRNA detection

RNA from mammalian cells was isolated using TRIzol reagents according to manufacturer protocol (Sigma, Catalog number- T9424) (Chomczynski and Sacchi, 1987; Zhang et al., 2018). Briefly, 500 ng of total RNA was converted to cDNA using the iScript cDNA synthesis kit (Thermo Fisher Scientific). RT–PCR (Bio-Rad) was performed with diluted cDNA (1:50) using SYBR Green master mix (Dynamo color flash SYBR Green qPCR kit, #F4167l) and primer sequences of the genes of interest, taking GAPDH as the housekeeping gene (Table 1). All samples were processed in technical triplicate and biological triplicate. ΔΔC_t_ value was calculated using the C_t_ values of the housekeeping gene and the NTCs.

**TABLE 1:** Different set of primer sequence used for expressional studies.

Sl. no.	Gene	Primer sequence
1.	*P53*	FP-5′ACCTATGGAAACTACTTCCTG3′
		RP-5′GAAGGGACAGAAGATGACA5′
2.	*BRCA1*	FP-5′CGCCTCAGCTCTGGTTGA3′
		RP-5′TGCCGCCAGCACGGACCGGT3′
3.	*BRIP1*	FP- 5′CCC​TCC​AGA​CAG​TTA​GGA​AT3′
		RP-5′GCTGGTTTCCCACTAAGAGA3′
4.	*BACH1*	FP-5′GATTAGCCTGGGCGATGATA 3′
		RF-5′ATGCATTTTCAGCAGGCTCT 3′
5.	*RAD50*	FP-5′CCATTATGCCGGAAGAAGAA3′
		RP-5′CAGTTTGCGGTTCAGTTCAA 3′
6.	*KU70*	FP-5′GAGCATCCAGTGTATCCAA3′
		RP-5′CAGCTTTAACCTGCTGAGT3′
7.	*GAPDH*	FP-5′AGGGCTGCTTTTAACTCTGGT3′
		RP-5′TCCCTCCAAAATCAAGTGGGG3′

### Western blotting

To isolate protein from the cells, Jang et al.’s (2019) protocol was followed. 50 ug of protein was loaded on 8%–10%-SDS PAGE depending on the size of the protein. The western blot analyses were performed as given by Gouda et al. (2018). The antibody details for all the proteins are listed in Supplementary Table S1.

### Cell cycle analysis

The cells (0.5 M cells/mL) were processed for flow cytometry according to the protocol given by Shen et al. (2017). Trypsinized cells were washed with 1X PBS and incubated with hypotonic DNA staining solution (0.5 mg/mL propidium iodide, 0.1% sodium citrate, and 0.05% Triton X-100) for 15 min at room temperature. The lysed cells were washed off from excess PI and analyzed by a Guava easyCyte flow cytometer (Merck, Millipore, FCS version 7.0) for the cell cycle. A total of 5,000 events were analyzed from each sample.

### Immunophenotyping

The cell surface marker CD24/44 and nuclear protein Ki67 expression were analyzed by immunophenotyping assay with the following protocol: 5 × 10^5^ cells were incubated with 500 ul of FACS staining buffer (FBS 2%, EDTA 0.5 M stock pH 8, 1X PBS and 1% Antibiotics) for 15 min and then washed with 1X PBS. The cell suspension was incubated with conjugated antibodies (Supplementary Table S1) at the concentration recommended by the manufacturer for 45 min at 4°C in the dark. The labeled cells were washed and re-suspended with FACS staining buffer. Overnight incubation was carried out for non-conjugated antibodies at 4°C, and secondary antibody incubation was performed for 45 min. The cells were washed with FACS staining buffer and analyzed using a Guava easyCyte flow cytometer.

### DAPI staining

For DAPI staining, 5 × 10^5^ cells were seeded on collagen-coated coverslips in each well of a six-well plate. At 80% confluency, the cells were washed with 1X PBS and fixed in 4% paraformaldehyde. Then, the cells were stained and mounted with PD-DAPI solution (0.5 μg/mL DAPI in 90% glycerol containing 1 mg/mL of the anti-fade dye *p*-phenylenediamine). The percentage of cells with heterogeneously stained DNA was carried out by quantitative analysis (counting ≥200 cells for each sample in *n* = 3 experiments).

### Immunofluorescence assay (IFA)

Immunofluorescence assay was performed for checking the BRCA1 and H2AX foci formation in cancer cells and in the cells which were treated with 0.035% MMS (methyl methane sulfonate) to check the repair activity using the following protocol: 5 × 10^4^ cells were seeded on collagen-coated coverslips in each well of a six-well plate. At 80% confluency, the cells were washed with 1X PBS and fixed in 4% paraformaldehyde, followed by permeabilization with 0.3% triton-X 100 in 1X PBS for 10 min each. Blocking was performed for 1 h with 5% FBS in 1X PBS at room temperature. The cells were incubated with primary antibodies overnight at 4°C in humid conditions, followed by the corresponding secondary antibody for 1 h at room temperature. After each antibody treatment, the cells were washed three times with 1X PBS. The coverslips containing cells were mounted on a glass slide with 10 ul of PD-DAPI, and imaging was carried out using an EVOS-M5000 microscope. For checking the repair activity, 0.035% MMS was added and incubated at 37°C for 15 min. After 15 min, the MMS was washed off with 1X PBS 2–3 times and released in fresh recommended culture media. The cells were fixed at 0 and 24 h after recovery. The cells without MMS were maintained as a control.

### Comet assay

Comet assay was performed as given by Olive and Banáth (2006), with slight modification. Cell pellets containing 5,000 cells were suspended in 0.8% low melting agarose in 0.9% saline solution, mixed well, and poured onto a frosted slide, and the coverslip was placed over the gel. After solidification, the slides were incubated overnight in freshly prepared lysis solution (2.5 M NaCl, 100 Mm Na. EDTA, 1% Triton-X 100, 10% DMSO) at 4°C. The slides were washed with freshly prepared alkaline buffer (300 Mm NaOH, 1 Mm NaEDTA, PH.13), followed by electrophoresis at 24 V for 30 min. The slides were washed with 0.4 M Tris-base and stained with EtBr solution. Imaging was carried out using ZOE fluorescent imager (Bio-Rad).

### DNA extraction and damage recovery by agarose gel electrophoresis

DNA from the different cell lines and the cells which were treated with 0.035% MMS was extracted by using the phenol-chloroform method. Briefly, for the fresh cell pellet, 875 ul of TE buffer was added and mixed well. For the same tube, 100 ul of 10% SDS and 1 mL of the phenol-chloroform mixture were added and incubated at room temperature for 5–10 min. After the centrifugation at 4°C for 10 min, the upper viscous supernatant was collected in a fresh tube. DNA precipitation was carried out by mixing it gently with 100 ul of 5 M sodium acetate. 2 mL of isopropanol was added and mixed by inversion until the white DNA strands were separated. The mixture was centrifuged, and the DNA was collected, followed by a 75% EtOH wash. The DNA pellet was dissolved in 1X TE buffer. The quantification was carried out using NanoDrop Colibri. 1–2 ug of DNA was loaded in 0.7% agarose and ran for around 22 h at 25 V and visualized under a gel documentation system to understand the movement of DNA in different cell lines. For damage recovery experiments, 0.035% MMS was added to the cells and incubated at 37°C for 15 min. After 15 min, the MMS was washed with 1X PBS 2–3 times, and the cells were released in fresh recommended culture medium. The cell pellet was collected at 0 h and after 24 h of recovery. The cells without MMS were maintained as a control.

### *In silico* data analysis

Gene expression data for TNBC, luminal type, and solid tissue normal data were downloaded from TCGA using UCSC Xena (Goldman et al., 2018). For phenotypes (N = 1,236) and gene expression, RNA seq data (N = 1,218) was downloaded as log2 (x+1)-transformed RSEM-normalized count values. The expression of the different genes in TNBC (N = 109), ER/PR^+ve^ (N = 371), and solid normal tissue (N = 119) were filtered. The association between gene expression of an individual gene and combinational genes was performed using R v3.6.3. A similar analysis was carried out with the dataset downloaded from cBioPortal. OS was analyzed only in the deceased population from UCSC Xena (N = 9) and cBioPortal (N = 15) concerning specific gene expression using Pearson’s correlation test. Data can be provided on request.

### Statistical analysis

All laboratory experiments were carried out minimum in biological triplicate and technical triplicates as and where required. Statistical analysis was performed using GraphPad Prism (v8.0). The gene expression was compared by one-way/two-way analysis of variance, followed by Tukey’s multiple comparisons. The unpaired *t*-test and Pearson correlation test were also performed when required. Potential outliers were identified using the ROUT method (Q = 10) implemented in GraphPad Prism (Motulsky and Brown, 2006). *p*-value <0.05 was considered statistically significant.

## Results

### Differential expression of TP53 and BRCA1 within triple-negative breast cancer groups

**FIGURE 1:**
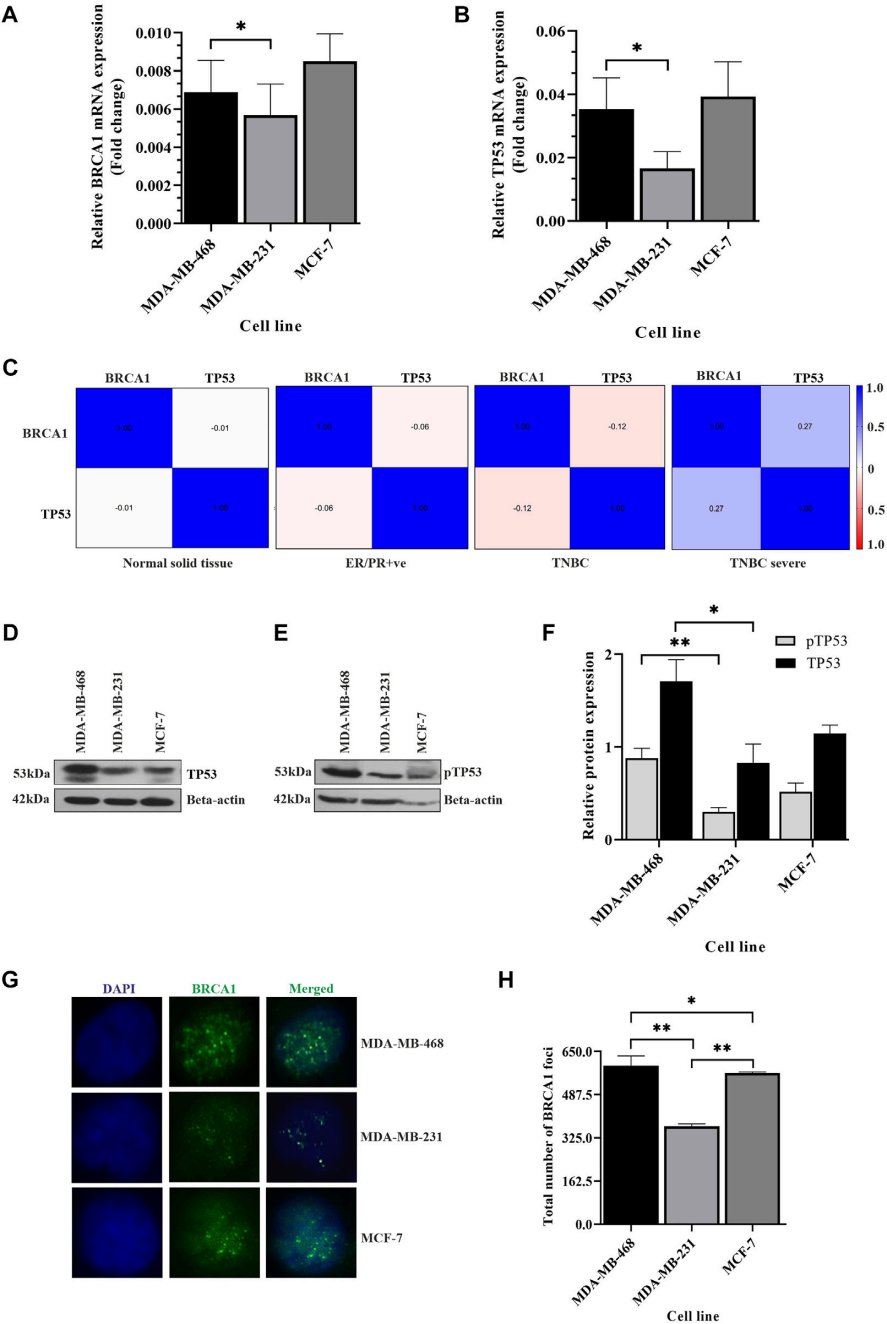
Differential expression of TP53 and BRCA1 within TNBC cells. **(A,B)** BRCA1 and TP53 expression changes with TNBC condition: The bar graph represents the expression studies performed with cDNA (500 ng of RNA) from different breast cancer lines, TNBC cell lines MDA-MB-468 and MDA-MB-231, and luminal type MCF-7. The expression was quantified from the technical triplicates of the RT qPCR data. **(C)** Association of BRCA1 and TP53 shows up in TNBC severe condition only: association between BRCA1 and TP53 in different BC tissues conditions obtained from TCGA data are analyzed by correlogram. Moving toward darker blue color indicates an increase in positive correlation, whereas toward darker red indicates an increase in negative correlation. The white color indicates no correlation. **(D,E)** TP53 and its functioning are compromised in some TNBC conditions only: TP53 expression, as well as its phosphorylation, was detected by western blot analysis of proteins extracted from the aforementioned cells, using corresponding (TP53 and phosphor-TP53) antibodies. β-Actin is given as the loading control. **(F)** Quantification of TP53 and pTP53 expression in breast cancer cells: the intensity of the TP53 and its phosphorylated bands from western blots were quantified using ImageJ software. The values of band intensities were normalized with the corresponding *β* -actin to normalize the protein loading. **(G)** Functioning of BRCA1 is also compromised in the MDA-MB-231 condition: functioning of the BRCA1 molecule is visualized by the formation of BRCA1 foci in all the different cell lines in the immunofluorescence assay. **(H)** Quantification of BRCA1-foci in TNBC cells: BRCA1-foci formation is scored in each cell and plotted as a bar graph. The *X*-axis represents the different cell lines, and *Y*-axis represents the total number of BRCA1 foci formed in 200 cells. All the experiments are performed in biological triplicates. Data were analyzed by one-way ANOVA, the Pearson correlation test, and the unpaired *t*-test (SEM is shown as error bars. *p < 0.05, **p < 0.005, and ^#^ *p* < 0.0001).

Clinical data suggest that TNBC cases with both *BRCA1* and *TP53* expression have better OS than cases that do not have their expression (Kim et al., 2016). When we analyzed the expression of the transcripts of BRCA1 and TP53 in different breast cancer groups, in the patient data taken from the TCGA database, we observed a significantly compromised expression of both the transcripts in the TNBC group, but we could not identify the variation in BRCA1 and TP53 expression within different patients of the TNBC group (*n* = 109) (Supplementary Figure S1A). The standard deviation of BRCA1/TP53 expression in different patients was only 0.14/0.13 from the mean value. The same compromised profile of BRCA1/TP53 was observed with the group of different TNBC cell lines with the luminal group (Supplementary Figure S1B). But, clinical findings suggest a differential prognosis of TNBC from patient to patient. So, there may be a differential expression of both the genes within triple-negative breast cancer conditions. To confirm this, we performed the expression studies of these genes in different TNBC lines: MDA-MB-231 and MDA-MB-468. Another breast cancer subtype cell line named MCF-7 was included in the experiments. As anticipated, we observed the difference in transcript levels of BRCA1 and TP53 in different lines of TNBC. The mRNA expression of both TP53 and BRCA1 were significantly compromised in MDA-MB-231 only, one TNBC condition, but not in MDA-MB-468, compared to the non-TNBC condition (MCF-7) (Figures 1A, B). Further analysis of the expressions of TP53 and BRCA1 in the different experimental cell lines revealed that there is no correlation between TP53 and BRCA1 in the case of ER/PR+ve condition (r = 0.393) and also in MDA-MB-468 TNBC cell line (r = −0.065). But in the case of MDA-MB-231, *BRCA1* correlated moderately with *TP53*, with both having less expression (r = 0.587). A similar distribution of correlation between the two genes was reflected in the *in silico* mRNA data analysis of the breast cancer data collected from the TCGA (r = −0.06 in ER/PR+ve group). Interestingly, depending on correlation, the TNBC patient sample group divides into two subgroups: TNBC mild condition (r = −0.12) and TNBC severe condition (deceased) (r = 0.274). MDA-MB-231 falls in the TNBC severe group (Figure 1C). So, the experimental observations with MDA-MB-231 fall in line with the *in silico* mRNA data analysis of the severe TNBC cases where a correlation between TP53 and BRCA1 develops.

To further reveal the effect of differential expression of BRCA1 and TP53, we studied the functioning of these transcripts in different TNBC cell lines. To understand the functional activity of TP53, we performed western blot analysis both for TP53 and phosho TP53. Whenever the DNA is challenged or integrity is compromised, the major checkpoint effector protein TP53 gets phosphorylated to activate the downstream mechanisms of cell cycle arrest and repair (Chen, 2016). We observed that TP53 is significantly overexpressed (*p* = 0.0293) in MDA-MB-468 than in MDA-MB-231. We also found compromised phosphorylation of TP53 in MDA-MB-231 compared to MDA-MB-468, *p* = 0.0424 (Figures 1D–F). To understand the functional activity of BRCA1, we analyzed the formation of BRCA1 foci at the damage sites, if there is any damage at all. BRCA1 is involved in the recognition and repair of aberrant structures in DNA and so, with an increase in damage, BRCA1 foci are formed (Wang et al., 2000). We observed the formation of BRCA1 foci to be significantly low in MDA-MB-231 than in MDA-MB-468(*p* = 0.0041) (Figures 1G, H). So, our results likely indicate that the robustness of checkpoint and repair proteins are compromised in one TNBC condition compared to the other and point toward differences in aggressiveness.

### The integrity of the DNA changes with different TNBC lines

**FIGURE 2:**
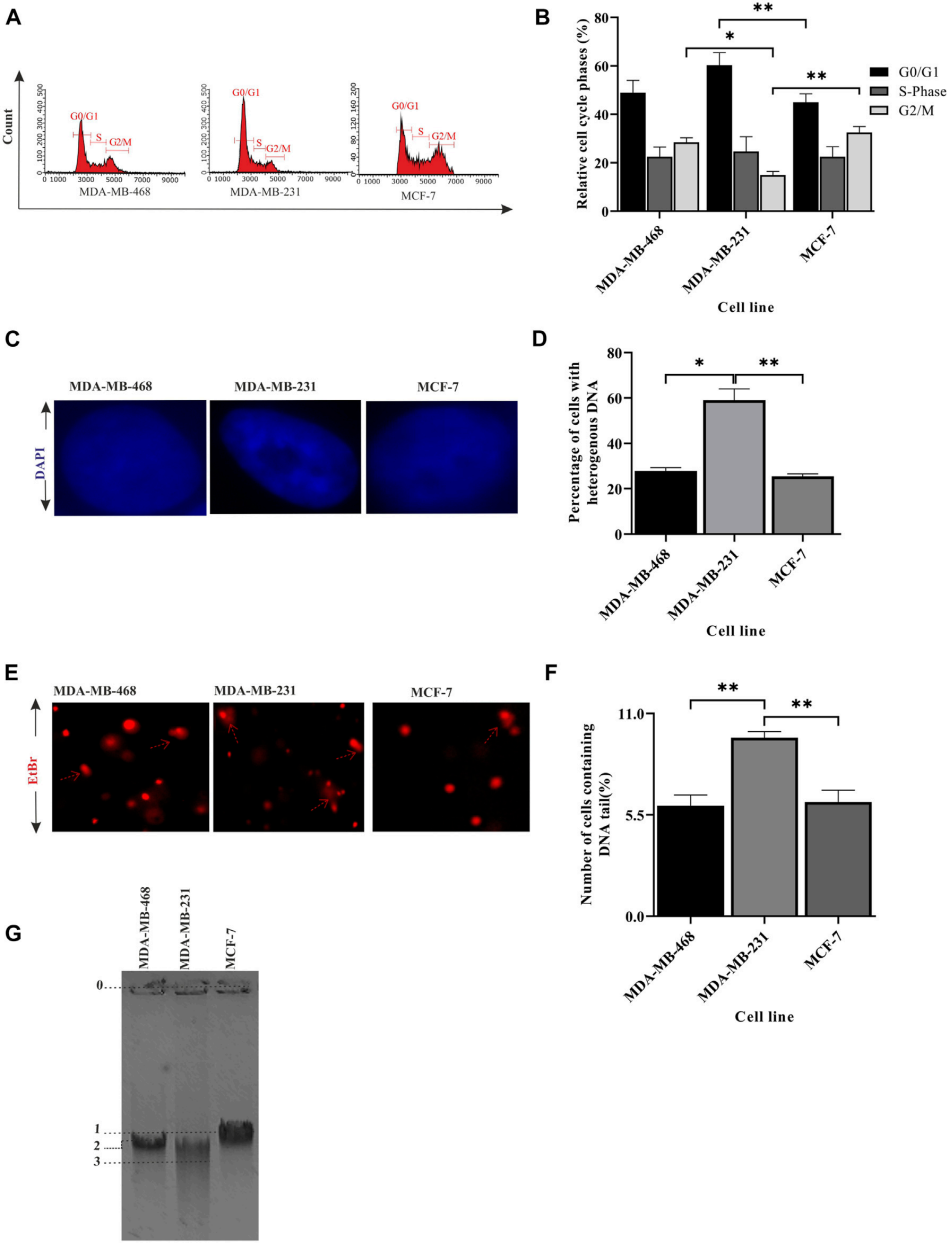
Integrity of the genetic material differs between TNBC lines. **(A)** MDA-MB-231 cells have a significantly less G2/M population: cell cycle analysis for different breast cancer cell lines, MDA-MB-468, MDA-MB-231, and MCF-7, was performed in a flow cytometer. The histogram plot represents the distribution of 5,000 cells into different phases of the cell cycle. **(B)** Quantification of the cell population at different stages: the population of cells in different phases of the cell cycle was analyzed and plotted in a bar graph using InCyte software of Guava easyCyte. **(C)** Heterogeneous arrangement of the genetic material in MDA-MB-231 cells suggests DNA damage: representative cell images (×100 magnification) of the aforementioned lines stained with DAPI show the homogeneous and heterogeneous arrangement of the genetic material. **(D)** Quantification of the increase in heterogeneity in the DNA of MDA-MB-231 cells: the bar graph represents the qualitative analysis of around 200 cells with heterogeneous (blue patches) and homogeneous (evenly spread blue) DNA. **(E)** Increase in tail formation in MDA-MB-231 confirms more DNA damage: representative image of cells having tails of fragmented DNA (comet) shows the extent of DNA damage in different cell types. The cells with the elongated tails are marked with broken red line arrows in the figure. **(F)** Quantification of the number of cells having tail DNA: the cells having comet (in all the fields captured) are plotted in the bar graph. A total of 150 cells were counted for each cell line. **(G)** The fragmented DNA in MDA-MB-231 cells moved the fastest through agarose gel electrophoresis: the figure represents the movement of DNA through agarose gel ran at low voltage for a longer time. The migrated distance from the starting point 0 to the mid of the DNA band marked as 1, 2, and 3 were measured. The distance traversed by MCF-7, MDA-MB-468, and MDA-MB-231 was 24.45, 25.40, and 29.90 mm, respectively. All the experiments were performed in biological triplicates. Data were analyzed by one-way ANOVA (SEM is shown as an error bar. *p < 0.05, **p < 0.005, ***p < 0.0005, and ^#^ *p* < 0.0001).

In the previous section, we showed compromised activity of the checkpoint pathway and HR-mediated repair due to improper BRCA1 foci formation in MDA-MB-231. So, we were interested in further observing the effect on the integrity of the DNA due to this condition and how significantly it varies within different TNBC lines. A perturbation in the genetic integrity shows a cell cycle arrest phenotype, so the cell cycle analysis was performed by staining the DNA content with PI (Figures 2A, B). We observed MDA-MB-231 cells to be mostly in G0/G1 and had a significantly low population of cells in the G2/M phase compared to MDA-MB-468 (*p* = 0.0095) and other cell types. This infers that it does not take much time at G2/M to maintain the genetic integrity and completes the cell cycle very fast because of the compromised checkpoint, reduced activity of TP53 (Figure 1F), and HR repair (through BRCA1) functions in MDA-MB-231 cells. On the contrary, the TNBC cell line MDA-MB-468 shows the same cell cycle profile as the luminal type, MCF-7, which suggests the proper functioning of the checkpoint and HR repair activity in MDA-MB-468 (Table 2). Furthermore, we checked for the integrity of the DNA by staining the cells with DAPI. We had observed the compact homogeneous arrangement of the nucleus in the case of MDA-MB-468 and MCF-7, but MDA-MB-231 nuclei were not compact, and the arrangement of the genetic material was found to be heterogeneous in nearly 54% of the cells compared to only 27% in MDA-MB-468 cells when analyzed in 200 cells (Figures 2C, D). To understand the heterogeneity in the DNA, we performed the comet assay and observed the formation of tails (Figure 2E). MDA-MB-231 cells showed high DNA damage, with 9.7% of cells containing tails of fragmented DNA, compared to MDA-MB-468, with only 6% of cells having fragmented DNA tails (*p* = 0.0035) (Figure 2F). In the case of MCF-7 cells, only 3% of the cells developed tails in the comet assay. The damage in the genetic material was also confirmed by performing a long-run low voltage agarose gel electrophoresis of the extracted DNA from these cell lines. We observed that MDA-MB-231 DNA moved fastest with a distance of 29.9 mm from the start point compared to the other TNBC line, MDA-MB-468, which was slower and traversed 25.4 mm only at the same time. MCF-7 DNA moved almost the same as MDA-MB-468, with a distance of 24.4 mm from the start point (Figure 2G). So, the increase in a smear in the MDA-MB-231 lane confirms the increase in the fragmentation and fast movement of the DNA compared to MDA-MB-468 DNA with a lighter smear.

**TABLE 2:** Cell cycle analysis of breast cancer cells.

Cells	Percentage in cell cycle		
	G0/G1	S-phase	G2/M
MDA-MB-468	49.37	15.941	33.823
MDA-MB-231	63.50	20.55	15.52
MCF-7	44.16	18.262	35.39

### DNA damage-sensing abilities change with different TNBC lines

**FIGURE 3:**
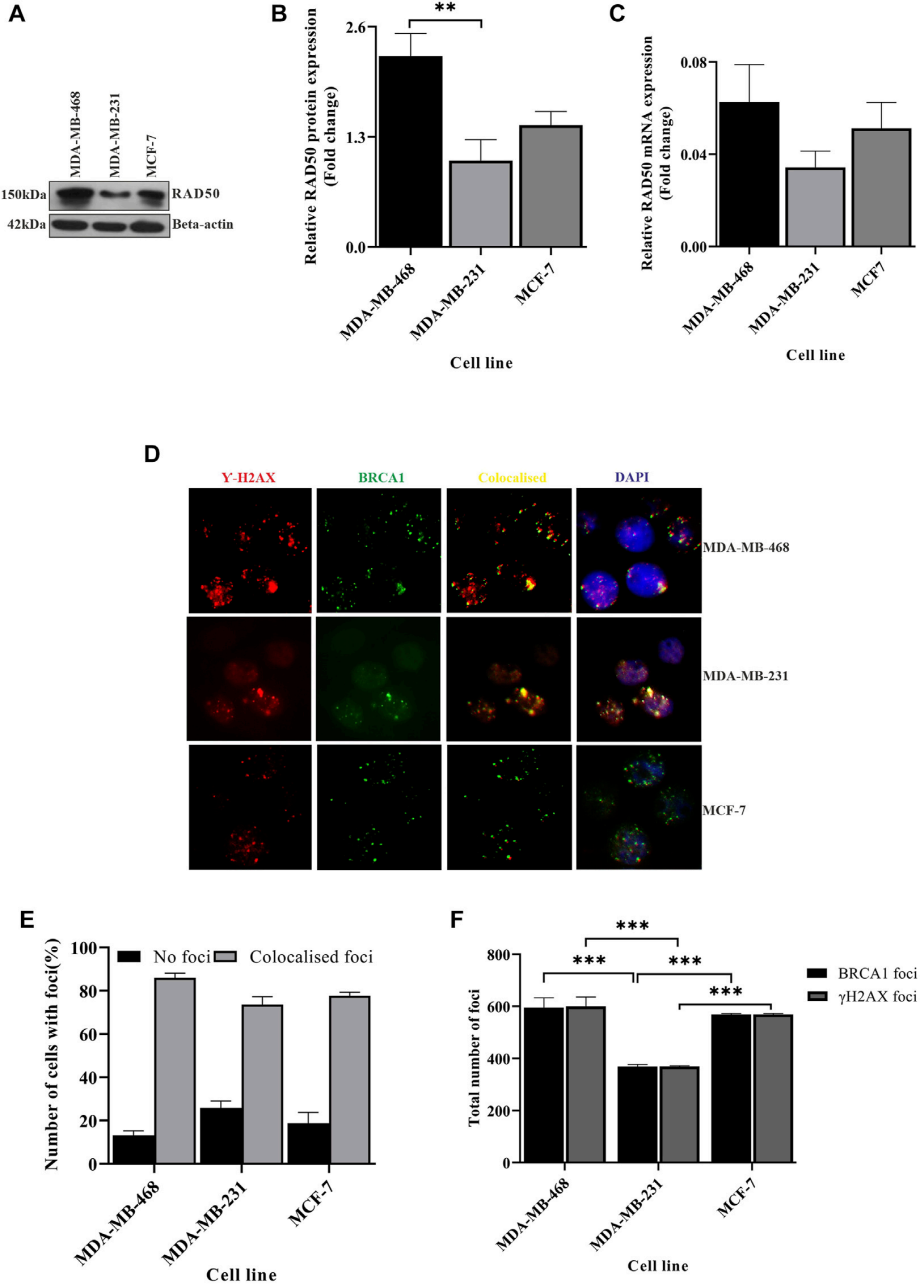
DNA damage sensing abilities changes between different TNBC lines. **(A)** Rad50 expression is compromised in MDA-MB-231 cells: RAD50 expression was detected by western blot analysis of proteins extracted from breast cancer lines, TNBC subtypes MDA-MB-468 and MDA-MB-231 and luminal type cell line MCF-7, using antibodies directed against Rad50 protein. *β* -Actin is considered as the loading control. **(B)** Quantification of RAD50 protein expression in all the cell types: the intensity of the RAD50 bands from the western blot analysis was quantified using ImageJ software. The values of band intensities were normalized with corresponding intensities of *β* -actin. **(C)** MDA-MB-231 cells showed compromised expression of RAD50 transcript also: expression studies were performed with cDNA (500 ng of RNA) from the aforementioned cell lines. The RAD50 expression was quantified by real-time PCR from the technical triplicates and represented in the bar graph. ∆∆Ct value was calculated from the C_t_ value of GAPDH and No template control (NTC). **(D)** Formation of less H2AX foci in MDA-MB-231: representative fields of immunofluorescence assay showing gamma-H2AX and BRCA1 foci. The red dots in the first panel indicate the gamma-H2AX foci, green dots in the second panel indicate the BRCA1 foci, yellow dots in the third panel indicate the co-localized foci, and blue dots in the fourth panel show the co-localization of H2AX-BRCA1 foci with the nucleus (DAPI). **(E)** The colocalization of BRCA1 with H2AX is mildly compromised in MDA-MB-231: the percentage of cells with no foci and co-localized foci was analyzed by counting 200 cells and is represented in the bar graph. **(F)** H2AX-BRCA1 foci as the marker for DNA damage sensing are significantly compromised in MDA-MB-231: the foci formation is analyzed manually by scoring the red and green dots in 200 cells. MDA-MB-231 with less H2AX BRCA1 foci confirms the defect in damage sensing. All the experiments in this figure were performed in biological triplicates. Data were analyzed by one-way and two-way ANOVA (SEM is shown as an error bar. *p < 0.05, **p < 0.005, and ***p < 0.0005).

We showed that the integrity of genetic material differs between different TNBC lines. The increase of DNA fragmentation in MDA-MB-231 cells leads to the accumulation of more DNA damage, but surprisingly, we saw compromised BRCA1 foci (Figure 1H) in MDA-MB-231 cells which developed at the damage sites. We, therefore, suspected an abrogation in DNA damage sensing capacity across the cell lines. So, we investigated the DNA damage sensing capacity in different BC lines and also in between the TNBCs by studying the DNA damage sensing molecules RAD50 and H2AX. Though we observed more DNA damage in MDA-MB-231, the expression of damage-sensing molecule RAD50 did not increase to the extent of DNA damage; instead, RAD50, both in the transcript and protein level, was downregulated in MDA-MB-231 cells than in MDA-MB-468 (Figures 3A–C). As RAD50 is low in MDA-MB-231, we further wanted to investigate the damage-sensing defect by observing the gamma-H2AX foci. H2AX binds to breaks and nicks of DNA molecules produced during any insult on DNA and forms damaged foci (Cannan and Pederson, 2016). Smaller foci are also formed during the mitotic phase (Cabrero et al., 2007). We counted the H2AX foci in approximately 200 cells in each of the different cell types (Figure 3D; Supplementary Table S2). We also analyzed the co-localization of the BRCA1-H2AX foci to confirm the specificity of BRCA1 foci to the sensors and justify the less number of BRCA1 dots in MDA-MB-231. We observed that the BRCA1 protein is associated with almost 98% of the H2AX protein in all the cell types but observed more cells having no foci in MDA-MB-231 (Figure 3E). Furthermore, in TNBC lines such as MDA-MB-231, more intense BRCA1-H2AX foci were formed significantly less in number compared to the other TNBC condition MDA-MB-468 cells (*p*-value 0.0001). The foci formation in MCF-7 was also significantly high compared to MDA-MB-231 cells (*p*-value 0.0003) because of a robust M-phase (Figures 3D, F). This suggests that due to compromised DNA damage-sensing molecule and shorter mitotic phase, the number of H2AX foci, as well as BRCA1 foci, is significantly less formed in MDA-MB-231 (Figures 3D, F). The absence of sensing the damage affects the downstream repair processes, which leads to the accumulation of more DNA damage compared to other TNBC subtypes and luminal subtypes (MCF-7). In conclusion, due to compromised sensing of DNA damage, the repair of the damage sites is also compromised leading to the accumulation of damage.

### Upregulation of the repair pathway due to accumulated damage

**FIGURE 4:**
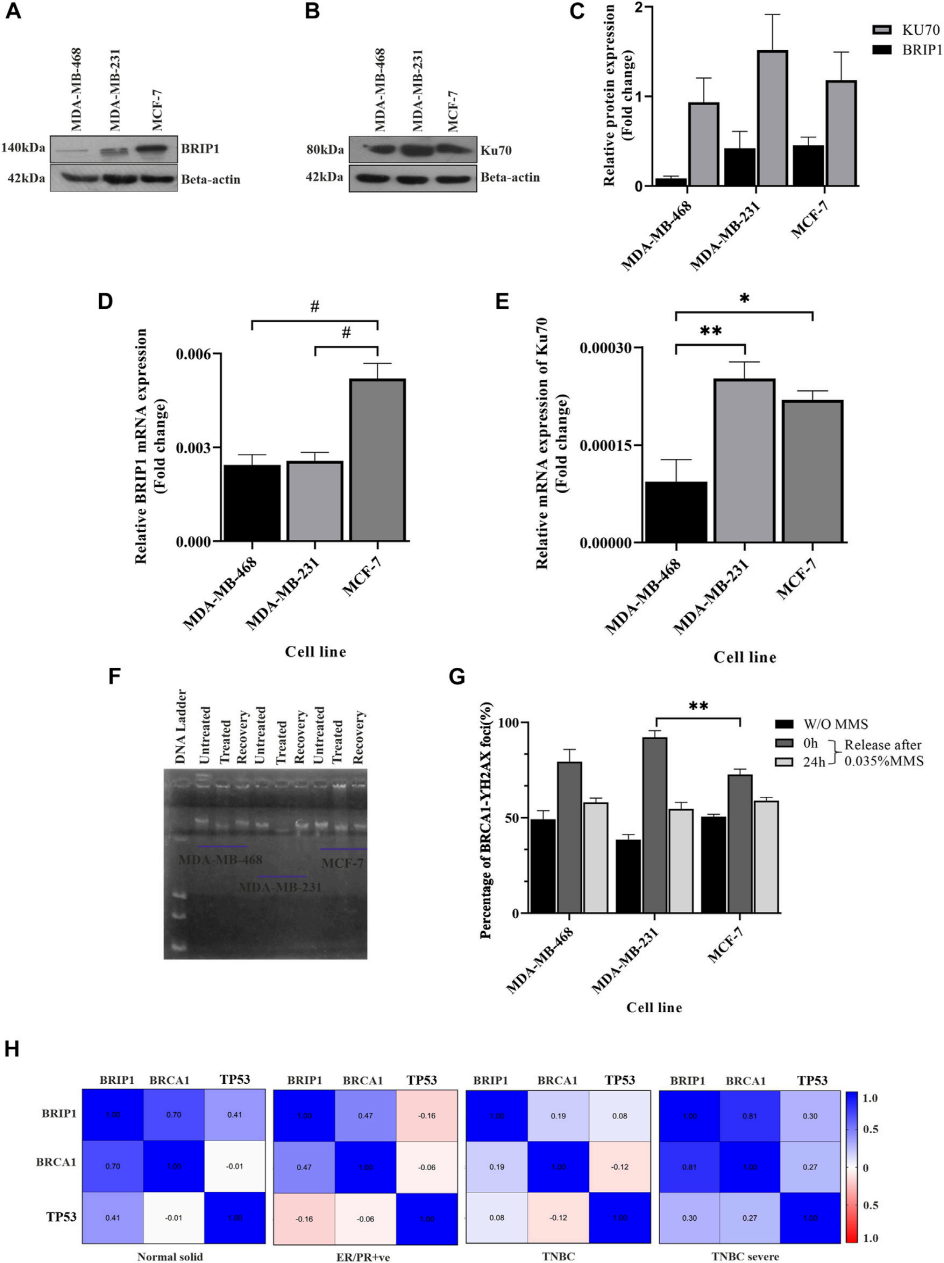
Upregulation of the repair pathway due to accumulated damage: **(A,B)** Overexpressed HR and NHEJ proteins in MDA-MB-231 cells. HR marker BRIP1 and NHEJ marker KU70 were detected by western blot analysis of proteins extracted from breast cancer lines, TNBC subtypes MDA-MB-468 and MDA-MB-231 and luminal type cell line MCF-7, using corresponding antibodies. *β* -Actin is given as the loading control. **(C)** Quantification of repair proteins in all the cell types. The intensity of the BRIP1 and KU70 bands from the western blot analysis was quantified using ImageJ software. The values of band intensities were normalized with corresponding intensities of *β* -actin. **(D)** The transcript level of BRIP1 is similar in TNBC cells: expression studies were performed with c DNA (500 ng of RNA) from the aforementioned lines. The bar graph represents the similar expression of BRIP1 in both the TNBC lines which were quantified by real-time qPCR from the technical triplicates and represented in the bar graph. **(E)** KU70 is overexpressed in MDA-MB-231 cells compared to other TNBC lines. The functional analysis of KU70 was performed with the extracted RNA from the aforementioned cell lines. The bar graph represents the expression of KU70 significantly more in the case of MDA-MB-231. The analysis was performed with technical triplicates. ∆∆Ct value was calculated from the Ct value of GAPDH, the housekeeping gene, and no template control (NTC), the non-specific background. **(F)** Robust DNA damage recovery in MDA-MB-231 due to the presence of overexpressed Ku70. The cells were treated with 0.035% MMS for 15 min and released in MMS-free media. The cells were harvested at 0 and 24 h, and the movement of DNA contents was visualized through agarose gel electrophoresis. The figure represents the movement of DNA through agarose gel run at 25 voltage for 22 h. **(G)** Percentage of BRCA1-H2AX foci formation in MMS-treated cells visualized by immunofluorescence assay. The foci formation is analyzed manually by scoring the green (BRCA1) and red (H2AX) dots in MMS untreated cells and 0 h/24 h post-MMS-treated cells (recovered). HR compromised cells MDA-MB-231 cells showed similar repair activity as HR efficient cells (MDA-MB-468) due to overexpressed NHEJ repair, justifying the robust repair. The *X*-axis represents the different cells, and the *Y*-axis represents the percentage of BRCA1-H2AX foci. **(H)** Association of BRCA1/TP53 with BRIP1 shows up only in TNBC severe condition. Association between BRCA1, TP53, and BRIP1 in different tissues condition like the normal solid sample (N = 119), ER/PR+ve (N = 314), TNBC (N = 95), and TNBC severe (N = 9) analyzed by UCSC-Xena (TCGA) is represented in correlogram. Moving toward darker blue color indicates an increase in positive correlation, whereas toward darker red indicates an increase in negative correlation. The white color indicates no correlation. All the experiments in this figure were performed in biological triplicates. Data were analyzed by one-way ANOVA and the Pearson correlation test (SEM is shown as error bar. *p < 0.05 and **p < 0.005).

In the previous sections, we showed that in some cases of TNBC such as MDA-MB-231, there is a completely compromised condition of the checkpoint, sensing of damage, and BRCA1 functioning, leading to accumulated damage. The pathways are relatively well-managed in another TNBC line: MDA-MB-468. Accumulated damage will lead to upregulated repair pathways. So, we further looked into the variations of the repair pathways in the two TNBC conditions. We checked the status of the repair molecules BRIP1, which repairs through homologous recombination, and Ku70, which follows the non-homologous end-joining (NHEJ) pathway. We observed that BRIP1 is slightly expressed more in MDA-MB-231 cells when compared to MDA-MB-468 cells, but the function is compromised as it does not reach the damage sites (Figures 4A, C, D) (less BRCA1 damage foci, Figures 1H, 3D). We also confirmed the over-functioning of the NHEJ pathway by showing that the expression of Ku70 in MDA-MB-231 cells is significantly high (Figures 4B, C, E). The overexpression of Ku70 and the presence of BRIP1 in MDA-MB-231 cells suggest the robustness of DNA repair. The robustness of repair is proved by the recovery experiments. We observed that greater damage in MDA-MB-231 is repaired similarly by 24 h, which is the same as in MDA-MB-468. The DNA moved faster in MDA-MB-231 after exposure to 0.035% MMS (methyl methane sulphonate), but it repaired to the same extent as MDA-MB-468 after recovery of 24 h (Figure 4F). The H2AX foci with damage increased in MDA-MB-231 and decreased drastically after recovery, confirming a robust repair despite less damage sensing through the HR pathway (Figure 4G; Supplementary Figure S2). A robust NHEJ repair in the presence of a compromised checkpoint, as shown in the previous section, happens in some TNBC conditions such as MDA-MB-231, which increases the chances of error-prone repair activity, followed by accumulated mutation (Figures 4B, C, E–G). The condition becomes mutation prone, which adds up to the complexity of tumorogenesis. To further correlate the role of these three proteins, namely, BRCA1, TP53, and BRIP1, in developing severe conditions, we performed the *in silico* correlation analysis of BRCA1 interacting repair molecule, BRIP1, with checkpoints in BC and TNBC patient groups collected from the TCGA database. In Figure 4H we found that though BRCA1 and BRIP1 correlate in all conditions, (normal, *P* = < 0.0001, r = 0.703; ER/PR+ve, *P*= <0.0001 r = 0.473 and TNBC, *p* = 0.049, r = 0.19), but there is no correlation between BRCA1 and TP53 (normal, *p* = 0.897, r = −0.012; ER/PR+ve, *p* = 0.3252 r = −0.056 and TNBC, *p* = 0.212, r = −0.121). However, in the case of severe TNBC condition, all three proteins are significantly correlated (BRCA1/BRIP1: *p* = 0.008, r = 0.813, BRCA1/*TP53*: *p* = 0.475, r = 0.274 and BRIP1/TP53:*p* = 0.438, r = 0.297). A similar correlation analysis was performed with the dataset collected from cBioPortal, and we found similar results (BRCA1/BRIP1: *p* = 0.003, r = 0.703, BRCA1/*TP53*: *p* = 0.644, r = 0.130 and BRIP1/TP53:*p* = 0.457, r = 0.208) (Supplementary Figure S3). So, this confirms that the correlation between the three genes develops only during severe TNBC conditions.

### Severity is highly dependent on upregulated repair and compromised checkpoint pathways

**FIGURE 5:**
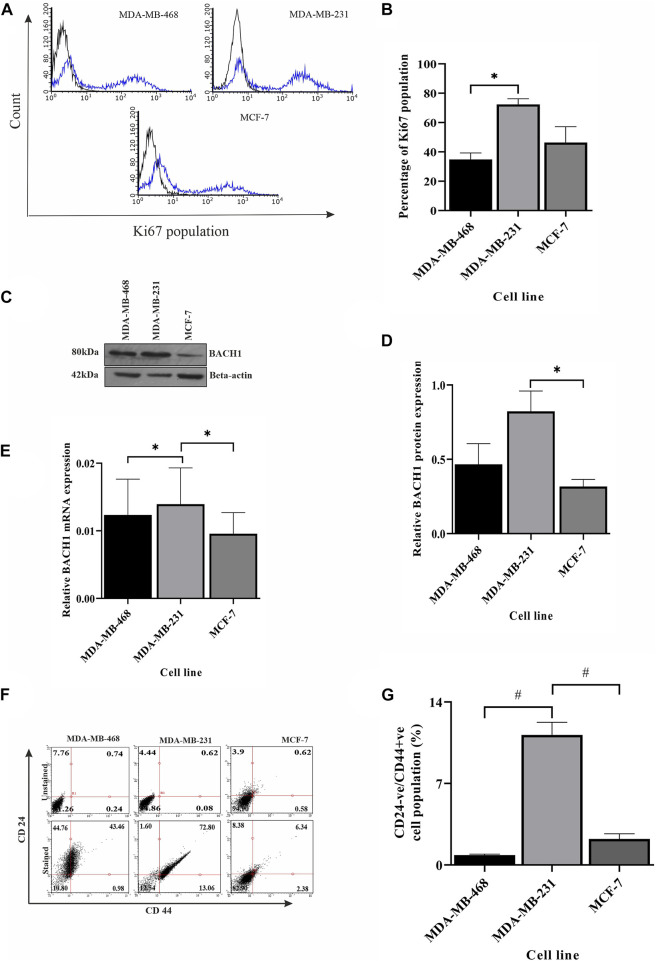
Severity is highly dependent on repair pathways. **(A)** Ki67 is overexpressed in MDA-MB-231 cells compared to other breast cancer subtypes. The proliferation of different breast cancer lines, TNBC subtypes MDA-MB-468 and MDA-MB-231, and luminal type cell line MCF-7 were monitored by measuring the Ki67 marker using immunophenotyping assay and represented by histogram plots (the black line indicates the unstained population, and the blue line indicates the Ki67 stained population). **(B)** Quantification of the Ki67 population in different cell types. The bar graph represents the percentages of cells having Ki67 expression, which was measured by gating the Ki67 population using Guava InCyte software. **(C)** The angiogenic/metastatic factor in breast cancer BACH1 overexpresses in MDA-MB-231 cells. The protein expression of metastasis marker BACH1 was detected by western blot analysis of proteins extracted from the aforementioned cells using the corresponding antibody. *β* -Actin is given as the loading control. **(D)** Quantification of BACH1 protein using ImageJ software. The bar graph represents the intensity of BACH1 bands from the Western blot analysis which was quantified using ImageJ software. The values of band intensities were normalized with corresponding intensities of *β* -actin. **(E)** MDA-MB-231 cells showed overexpression of BACH1 transcript also: expression studies were performed with cDNA (500 ng of RNA) from the aforementioned cell lines. The BACH1 expression was quantified by RT-PCR from the technical triplicates and represented in the bar graph. ∆∆Ct value was calculated from the C_t_ value of GAPDH and no template control (NTC). **(F)** MDA-MB-231 has more cancer stem cell population. The cancer stem cell population was analyzed by immunophenotyping assay. The dot plots represent the CD44+ve/CD24-ve stained (upper panel) and unstained (lower panel) population in different cell types. **(G)** Quantification of the higher population of cancer stem cells in MDA-MB-231: The bar graph represents the percentages of cells having CD44+ve/CD24-ve expression, which was measured by gating the CD44+ve/CD24-ve population using Guava InCyte software. All the experiments were performed in biological triplicates. Data were analyzed by one-way ANOVA (SEM is shown as error bar. *p < 0.05 and **p < 0.005).

So far, we showed the development of different conditions in different TNBC cell lines. Correlation analysis between BRCA1, BRIP1, and TP53 justifies the development of a condition where there is upregulated NHEJ repair in the absence of robust checkpoint and sensing of damage. This condition is prone to mutations (Chatterjee and Walker, 2017). Accumulated mutations will add up to the complexity of tumorogenesis. In this final section, we identified that the conditions developed in MDA-MB-231 developed severity compared to the mild conditions in MDA-MB-468. One of the hallmarks of severity is proliferation. In Figures 5A, B, we have shown through immunophenotyping that more than 72% of the cells contain the proliferation marker, Ki67, in the case of the MDA-MB-231 cell compared to only 34.86% and 46.40% of cells in MDA-MB-468 and MCF-7, respectively (Figures 5A, B). We also looked at the expression of the transcription factor, BTB and CNC homology 1, BACH1, which has a role in metastasis, angiogenesis, and development of aggressive behavior of breast cancer cells (Zhang et al., 2018). The expression of BACH1 protein was significantly more in MDA-MB-231 cells (Figures 5C, D). We further analyzed the transcript level of BACH1 in all the cell lines and confirmed that the expression of BACH1 is significantly more in MDA-MB-231 than in the rest of the lines (*p* = 0.0295, Figure 5E). We also looked for the stemness property of the different BC groups, as well as in different TNBC cells, by quantifying cancer stem cell population through immunophenotyping. Figures 5F, G shows that there is a higher population of CD44^+^/CD24^-^ cells in MDA-MB-231 (13.06%) than in MDA-MB-468 (0.98%) and other breast cancer line MCF-7 (2.38%) (Figures 5F, G). So, in this section, we proved that MDA-MB-231 cells show more stemness and metastatic properties than MDA-MB-468. To further support that the condition developed by correlation of BRCA1/BRIP1/TP53 in MDA-MB-231 is severe, we analyzed the OS in TCGA patient data with the concerning proteins. The data show that the expression of individual transcript (BRCA1 or BRIP1 or TP53) does not correlate with OS in the case of ER/PR+ve, but in the case of TNBC, OS is associated with the overexpressed BRCA1 (*p* = 0.0272, R^2^ = 0.5254) (Table 3). Surprisingly, when the expression of BRIP1 is considered along with BRCA1 and analyzed for OS, only TNBCs have a significant increase in association with BRCA1/BRIP1 expression and OS (*p* = 0.0008706, R^2^ = 0.9045). Finally, when expression of all three genes (BRCA1, BRIP1, and *TP53*) is considered for their correlation with OS, a synergistic association with OS was observed over BRCA1/BRIP1 in TNBC patients only (*p* = 0.0055, R^2^ = 0.9048) (Table 3).

**TABLE 3:** Association of the m-RNA expression of *BRCA1*, *BRIP1,* and *TP53* genes with respect to OS in TNBC and ER/PR^+VE^ breast cancer.

Subtypes→	ER^-ve^/PR^-ve^/HER2^-ve^		ER^+ve^/PR^+ve^/HER2^-ve^	
Expression↓	R2	*p*-value	R2	*p*-value
*BRCA1* vs*. BRIP1*	0.6616	0.0077**	0.8839	<0.0001****
*BRIP1* vs. *TP53*	0.08796	0.4384	0.003329	0.8662
*BRCA1* vs. *TP53*	0.5349	0.1006	0.005288	0.8317
OS vs. *BRIP1*	0.05351	0.5493	0.01227	0.7458
OS vs. *BRCA1*	0.5254	0.0272*	0.002304	0.8885
OS vs. *TP53*	0.01107	0.7876	0.01760	0.6974
*BRCA1* + *BRIP1* vs. OS	0.9045	0.0008706**	0.03939	0.8515
*BRIP1* + *TP53* vs. OS	0.05498	0.844	0.03167	0.8792
*BRCA1* + *TP53* vs. OS	0.5349	0.1006	0.02094	0.9188
*BRCA1* + *TP53* + *BRIP1* vs. OS	0.9048	0.0055**	0.005643	0.9332

*- *p* Value <0.05, **- *p* Value <0.01, ****- *p* value <0.0001.

## Discussion

TNBC shows aggressive behavior, having a high rate of metastasis and an increased re-occurrence rate. Also, its prognosis is poor due to the lack of effective therapy (Stolarova et al., 2020). In addition to all the complexities of this disease, its characteristics vary from one patient to another patient, which has baffled clinicians. In this study, we addressed the likely reason behind the differential characteristics of TNBC cases. We studied the molecules and the mechanisms involved in TNBC progression/re-occurrence/metastasis and revealed the molecular factors that play a role in determining the variation in severity in TNBC. When 109 patient samples with TNBC condition were considered as a group, we observed a loss of expression of TP53 and BRCA1, compared to ER/PR+ve breast cancer. However, the expression profile of the same genes in different TNBC cell lines does not follow the analysis performed on publicly available data. From our *in vitro* experiments, we observed that in some TNBC lines such as MDA-MB-231, the reduction in the expression of BRCA1 and TP53 is more compared with other triple-negative and ER/PR+ve breast cancer. However, in some, such as MDA-MB-468, the expression of BRCA1/TP53 is discernibly higher than in the ER/PR+ve condition. The formation of BRCA1 foci and phosphorylated TP53 was less in MDA-MB-231, insinuating the compromised functioning of the genes. The reduced functioning of the TP53 checkpoint allows the cells to move fast in the G2-phase, taking less time to rectify the damage, which affects the integrity of the DNA more in MDA-MB-231 cells. This was evident from the fast movement of DNA through agarose gel and tails in the comet assay. But surprisingly, the sensing of damage by RAD50, followed by the formation of γ-H2AX foci, was significantly compromised in MDA-MB-231 cells. Less phosphorylated H2AX results in an unstable DDR complex at the damaged sites which, in turn, leads to impaired error-free HR activity through the BRCA1–BRIP1 axis (Krum et al., 2010; Zimmermann and de Lange, 2014). In the case of MDA-MB-468, the MRN complex is more active, as evident from increased RAD50 expression. As a result, the number of H2AX followed by BRCA1 foci was more compared to the other TNBC line MDA-MB-231 and preferred repair of damaged DNA by HR. So, in some cases of TNBC, the damage is more due to compromised BRCA1, and the processing of the damage is also improper because of the absence of the other interacting proteins of BRCA1 such as RAD50.

Abrogation in checkpoints leads to the accumulation of more damage which, in turn, promotes more repair activity, as evident from the observed expression of the BRIP1 and overexpression of Ku70 in MDA-MB-231. Overexpression of two arms of the DNA repair pathway simultaneously in TNBC cell lines, independent of BRCA mutation status, resulted in irreparable DNA damage and subsequent cell death (Alshareeda et al., 2013; Boerner et al., 2015). As BRIP1 is unable to reach the functional site due to the absence of RAD50 (compromised BRCA1 function) followed by γ-H2AX, so to promote repair to the accumulated damage, the cells follow the NHEJ repair pathway resulting in DNA repair with accumulated mutations (Chiruvella et al., 2013). An increase in mutations leads to more aggressive tumors (Takahashi et al., 2020). The markers of aggressive tumors were observed more in the case of MDA-MB-231 cells with higher Ki67 expression (Blagosklonny, 2006). Ma et al. (2014) have shown that the CD44+/CD24− cell population with cancer stem cell-like properties may play an important role in the aggressive behavior of TNBC. We also confirmed the increase in the stem-ness property of MDA-MB-231 cells, as more than 70% of the population of cells contain the cancer stem cell marker compared to the other TNBC (40%) and ER/PR^+ve^ BC (6%) cell types. We also observed the upregulated expression of BACH1 in MDA-MB-231 cells. BACH1 is a transcription factor having a role in tumor relapse and metastasis in breast cancer (35). So, from the abovementioned experiment, we conclude that MDA-MB-231 has the worst prognosis with higher chances of severity, aggressiveness, and metastasis than the MDA-MB-468 and MCF-7 cell lines, an observation that is also in line with clinical observations.

Analysis of OS with the publicly available patient data confirms the same correlation of BRCA1, TP53, and BRIP1 with severity. The data show that there is a correlation between BRCA1 and BRIP1 in breast cancer patients (Table 3). The expression of BRCA1 and BRIP1 is associated with the luminal type (*p* = 0.0001) and TNBC (*p* = 0.0077), but the OS is related with BRCA1 with a correlation of R^2^ = 0.525 only in the case of TNBC (*p* = 0.0272) (Table 3). The correlation of OS with BRCA1 in TNBC increases (R^2^ = 0.9045, *p* = 0.00087) when BRIP1 is considered along with BRCA1. So, the decrease in overall survivability or increase in severity depends on the interplay of both the proteins BRCA1 and BRIPI and also the combination of three proteins, i.e., BRCA1, BRIP1, and TP53 (*p* = 0.0055) in certain TNBC conditions. These data highly support our experimental data, where we have concluded in the previous section that less functioning of BRIP1, due to compromised BRCA1 activity, leads to the severity with overexpression of proliferative, stemness, angiogenic, and metastatic markers, supporting the aggressive nature of cancer progression.

Thus, we have partially defined the signaling cascade of repair molecules and established it for the development of severity in TNBC. We identified the direct association of the DNA repair pathway with the severity of breast cancer. This is supported by the fact that compromised checkpoint and less functional BRCA1 leads to more accumulated damage. Also, low sensing of damage through RAD50 and H2AX takes place, leading to less BRCA1 at the damage sites. Less BRCA1 at damage sites leads to less error-free repair through BRIP1, while the co-expression of BRCA1/TP53 in some TNBC conditions supports the efficient repair activity through HR with less chance of recurrence and severity. Furthermore, high expression of Ku70 leads to error-prone NHEJ repair activity in BRCA1/TP53 compromised cases, developing the chance of mutations at the molecular level. Overexpressed Ku70 lead to tumorogenic conditions, which can be visible by more tumorogenic phenotypes. Hence, understanding the breast cancer subtypes is essential in terms of personalized medicine. Our study sheds light on the aggressiveness of one of the subtypes of breast cancer which happens as a result of a lack of efficient repair activity due to compromised sensing of DNA damage and promotion of error-prone repair activity.

## Data Availability

The original contributions presented in the study are included in the article/Supplementary Material, further inquiries can be directed to the corresponding author.
