# Supplementary figures and images for: BRCA1 interactors, RAD50 and BRIP1, as prognostic markers for triple-negative breast cancer severity
Source: Front Genet. 2023 Feb 16;14:1035052. doi: 10.3389/fgene.2023.1035052 (PMC9978165; doi:10.3389/fgene.2023.1035052)

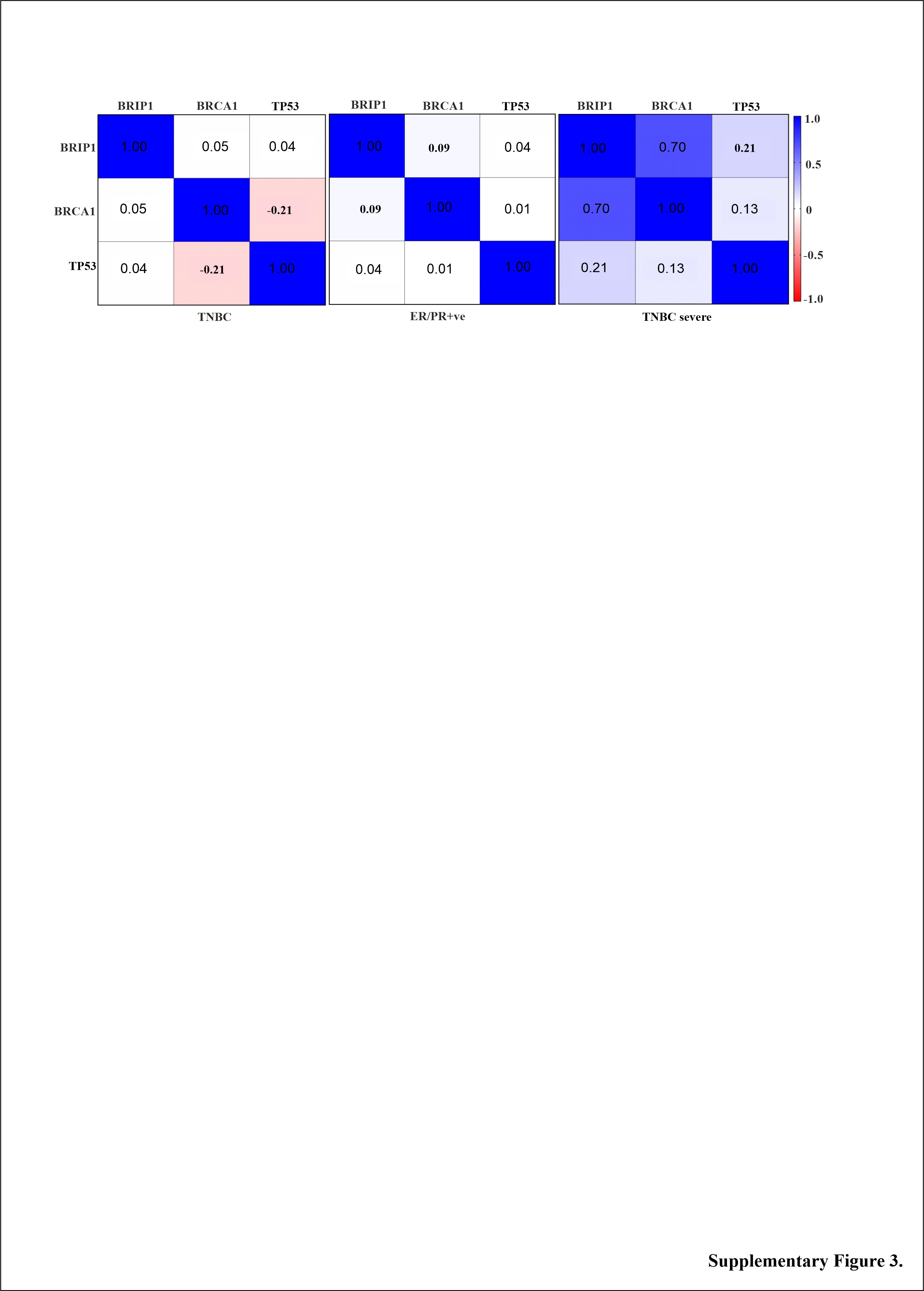

Supplement: Supplementary file 1 [file Image3.JPEG]

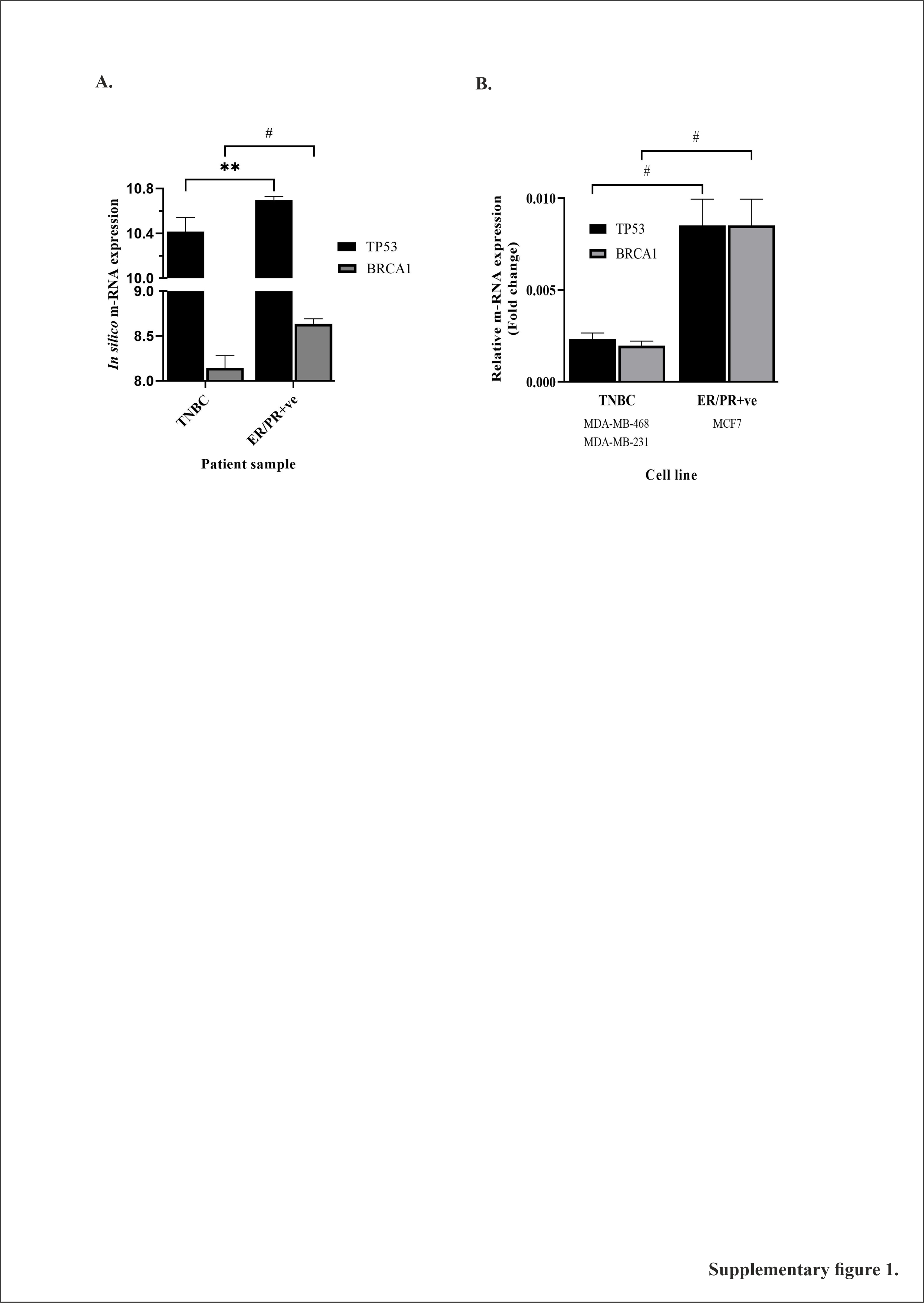

Supplement: Supplementary file 3 [file Image1.JPEG]

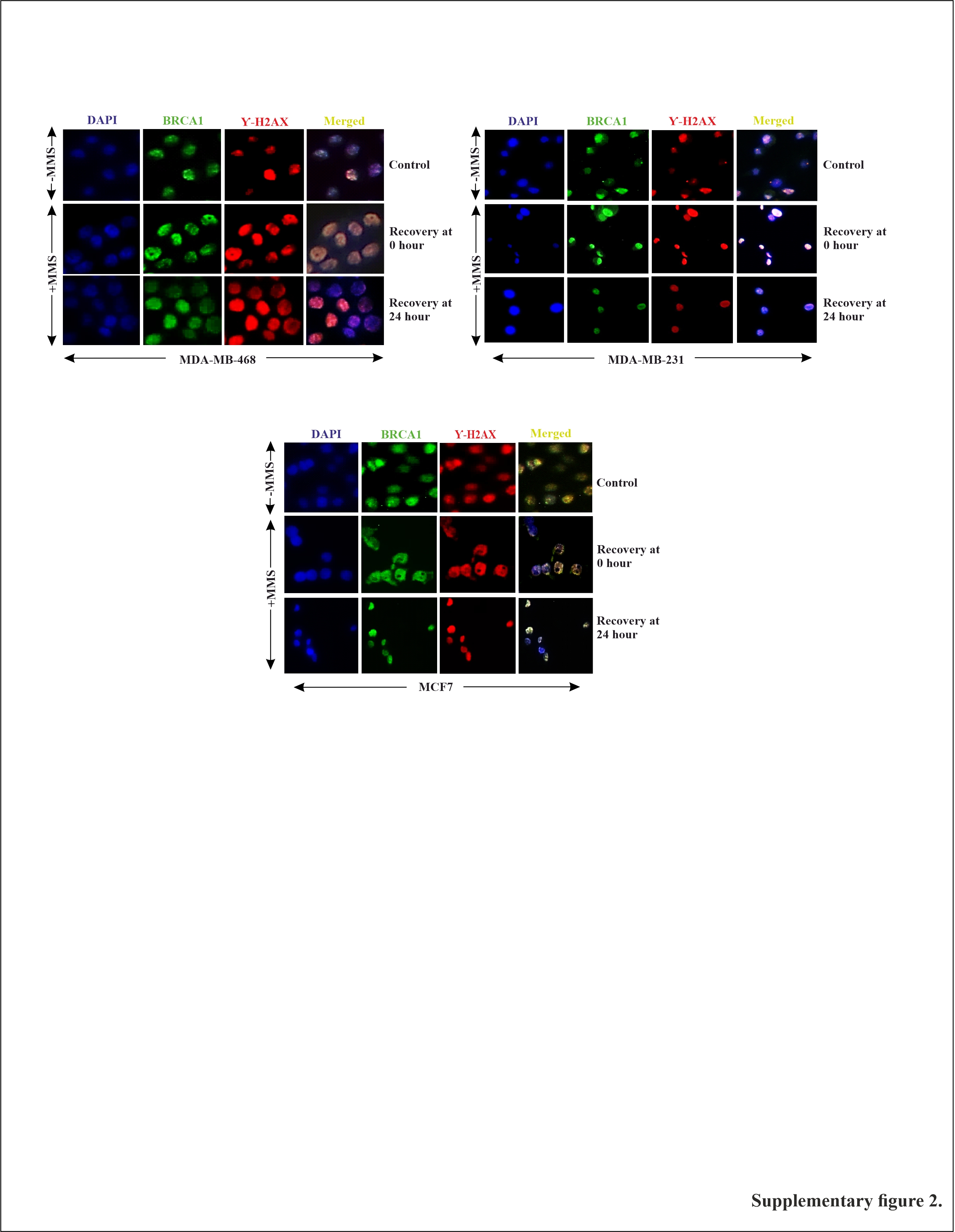

Supplement: Supplementary file 4 [file Image2.JPEG]
