# Supplementary material for: BRCA1 interactors, RAD50 and BRIP1, as prognostic markers for triple-negative breast cancer severity
Source: Front Genet. 2023 Feb 16;14:1035052. doi: 10.3389/fgene.2023.1035052 (PMC9978165; doi:10.3389/fgene.2023.1035052)
Supplement: Supplementary file 2 [file Table1.pdf]

**Supplementary Table 1. Antibody used in different experiments:**

**(western blot, Immuno-fluorecence and Immuno-phenotyping)**

| Sl. No. | Name of the protein | 1° antibody details | 2° antibody details |
|---|---|---|---|
| **1.** | BRCA1 | BRCA1, cat. no. MA1-23164 Thermo scientific | Anti mouse, cat. no.7074, CST |
| **2.** | TP53 | TP53 , CST | Anti rabbit, cat. no.7074, CST |
| **3.** | BRIP1 | BRIP1, cat. no.4578, CST | Anti rabbit, cat. no.7074,CST |
| **4.** | BACH1 | BACH1, cat. no.sc-271211, Santa Cruz | Anti mouse, cat. no.7076,Cloud clone |
| **5.** | KU70 | KU70, cat. no. MA5-13110, Thermo scientific | Anti mouse, cat. no.7076, CST |
| **6.** | RAD50 | RAD50, cat. no.PAK11HU01, Cloud clone | Anti rabbit, cat. no. 7074, CST |
| **7.** | Phospho-TP53 | Phospho-p53 Antibody, cat. no. 9284, CST | Anti rabbit, cat. no. 7074, CST |
| **8.** | Beta Actin | Beta Actin, cat. no. 4967, CST | Anti rabbit, cat. no.7074,CST |
| **9.** | Ki67 | Ki67, cat. no. PA5-16785, Thermo scientific | Goat Anti rabbit IgG, Dy-Light 488, Thermo scientific |
| **10.** | Phospho-Histone H2AX | Phospho-Histone H2AX, cat. no.MA1-2022, Thermo scientific | Goat anti mouse IgG, Dy-light594, Thermo scientific |
| **11.** | CD44 | Anti-CD44 antibody (PE/Cy7), cat. no. ab46793, Abcam | Conjugated |
| **12.** | CD24 | Anti-CD24 antibody(FITC), cat. no. ab30350, Abcam | Conjugated |
