# Supplementary material for: BRCA1 interactors, RAD50 and BRIP1, as prognostic markers for triple-negative breast cancer severity
Source: Front Genet. 2023 Feb 16;14:1035052. doi: 10.3389/fgene.2023.1035052 (PMC9978165; doi:10.3389/fgene.2023.1035052)
Supplement: Supplementary file 5 [file Table2.docx]

**Supplementary table 2:** Analysis of BRCA1-ƳH2AX foci in MDA-MB-468, MDA-MB-231 and MCF cell line through Immunofluorescence assay.

| **Cells** | **Cells having BRCA1 foci(%)** | | **Cells having H2ax foci** | | **Colocalised foci** | | **Non colocalised**  **foci** |
| --- | --- | --- | --- | --- | --- | --- | --- |
| **468** | **1-5 foci** | **More foci(Number of foci)** | **1-5 foci** | **More foci(Number of foci)** | **1-5 foci** | **More foci(Number of foci)** |  |
| 1 | 5 |  | 5 |  | 5 |  |  |
| 2 |  | 11 |  | 11 |  | 11 |  |
| 3 | 2 |  | 2 |  | 2 |  |  |
| 4 |  | 11 |  | 11 |  | 11 |  |
| 5 |  | 15 |  | 15 |  | 15 |  |
| 6 | 5 |  | 5 |  | 4 |  | 1 |
| 7 |  | 7 |  | 7 |  | 7 |  |
| 8 |  | 8 |  | 8 |  | 8 |  |
| 9 |  | 7 |  | 7 |  | 6 | 1 |
| 10 | 2 |  | 2 |  | 3 |  |  |
| 11 |  | 9 |  | 9 |  | 9 |  |
| 12 | 5 |  |  | 6 |  | 6 |  |
| 13 |  | 8 |  | 8 |  | 8 |  |
| 14 | 2 |  | 2 |  | 2 |  |  |
| 15 |  | 7 |  | 7 |  | 7 |  |
| 16 | 2 |  | 2 |  | 2 |  |  |
| 17 |  | 11 |  | 11 |  | 11 |  |
| 18 | 4 |  | 4 |  | 4 |  |  |
| 19 | 0 |  |  | 1 |  |  | 1 |
| 20 |  | 6 |  | 6 |  | 6 |  |
| 21 | 3 |  | 3 |  | 3 |  |  |
| 22 |  | 7 |  | 7 |  | 7 |  |
| 23 |  | 7 |  | 7 |  | 7 |  |
| 24 |  | 8 |  | 9 |  | 8 | 1 |
| 25 | 0 | 9 |  | 10 |  | 9 | 1 |
| 26 |  | 7 |  | 7 |  | 7 |  |
| 27 |  | 8 |  | 8 |  | 8 |  |
| 28 |  | 11 |  | 11 |  | 11 |  |
| 29 |  | 6 |  | 6 |  | 6 |  |
| 30 | 2 |  | 2 |  | 2 |  |  |
| 31 |  | 9 |  | 9 |  | 9 |  |
| 32 |  | 12 |  | 12 |  | 12 |  |
| 33 |  | 8 |  | 8 |  | 8 |  |
| 34 | 1 |  | 1 |  | 1 |  |  |
| 35 | 4 | 0 | 4 |  | 4 |  |  |
| 36 | 3 |  | 3 |  | 3 |  |  |
| 37 | 4 |  | 4 |  | 4 |  |  |
| 38 |  | 8 | 0 | 8 | 0 | 8 |  |
| 39 | 1 |  | 1 |  | 1 |  |  |
| 40 |  | 8 |  | 8 |  | 8 |  |
| 41 | 3 |  | 3 |  | 3 |  |  |
| 42 |  | 8 |  | 8 |  | 8 |  |
| 43 |  | 7 |  | 7 |  | 7 |  |
| 44 |  | 10 |  | 10 |  | 10 |  |
| 45 |  | 7 |  | 6 |  | 6 | 1 |
| 46 |  | 8 |  | 8 |  | 8 |  |
| 47 |  | 11 |  | 11 |  | 11 |  |
| 48 |  | 12 |  | 12 |  | 12 |  |
| 49 |  | 6 |  | 6 |  | 6 |  |
| 50 | 1 |  | 1 |  | 1 |  |  |
| 51 | 3 |  | 3 |  | 3 |  |  |
| 52 | 1 |  | 1 |  | 1 |  |  |
| 53 |  | 8 | 0 | 8 |  | 8 |  |
| 54 | 5 |  | 5 |  | 5 |  |  |
| 55 | 0 | 11 |  | 11 |  | 11 |  |
| 56 | 3 |  | 3 |  | 3 |  |  |
| 57 | 4 |  | 4 |  | 4 |  |  |
| 58 |  | 12 |  | 12 |  | 12 |  |
| 59 |  | 6 |  | 6 |  | 6 |  |
| 60 |  | 7 |  | 7 |  | 7 |  |
| 61 |  | 7 |  | 7 |  | 7 |  |
| 62 |  | 8 |  | 8 |  | 8 |  |
| 63 |  | 9 |  | 9 |  | 9 |  |
| 64 | 5 |  | 5 |  | 5 |  |  |
| 65 | 3 |  | 3 |  | 3 |  |  |
| 66 |  | 6 |  | 6 |  | 6 |  |
| 67 |  | 8 |  | 8 |  | 8 |  |
| 68 |  | 11 |  | 11 |  | 11 |  |
| 69 | 5 | 0 | 5 |  | 5 |  |  |
| 70 | 0 | 11 |  | 11 |  | 11 |  |
| 71 | 1 |  | 1 |  | 1 |  |  |
| 72 | 0 |  | 0 |  | 0 |  |  |
| 73 |  | 6 |  | 6 | 0 | 6 |  |
| 74 |  | 7 |  | 7 |  | 7 |  |
| 75 | 3 |  | 3 |  | 3 |  |  |
| 76 |  | 12 |  | 12 |  | 12 |  |
| 77 | 3 |  | 3 |  | 3 |  |  |
| 78 | 5 |  | 5 |  | 5 |  |  |
| 79 |  | 6 |  | 6 |  | 6 |  |
| 80 |  | 8 |  | 8 |  | 8 |  |
| 81 | 3 |  | 3 |  | 3 |  |  |
| 82 |  | 8 |  | 8 |  | 8 |  |
| 83 |  | 10 |  | 10 |  | 10 |  |
| 84 |  | 6 |  | 6 |  | 6 |  |
| 85 | 5 |  | 5 |  | 5 |  |  |
| 86 |  | 7 |  | 7 |  | 7 |  |
| 87 |  | 6 |  | 6 |  | 6 |  |
| 88 | 2 |  | 2 |  | 2 |  |  |
| 89 | 3 |  | 3 |  | 3 |  |  |
| 90 |  | 11 |  | 11 |  | 11 |  |
| 91 | 3 |  | 3 |  | 3 |  |  |
| 92 |  | 10 |  | 10 |  | 10 |  |
| 93 | 1 |  | 1 |  | 1 |  |  |
| 94 |  | 6 |  | 6 |  | 6 |  |
| 95 |  | 8 |  | 8 |  | 8 |  |
| 96 |  | 9 |  | 9 |  | 9 |  |
| 97 | 1 |  | 1 |  | 1 |  |  |
| 98 |  | 7 |  | 7 |  | 7 |  |
| 99 |  | 6 |  | 6 |  | 6 |  |
| 100 |  | 11 |  | 11 |  | 11 |  |
| Total | 108 | 525 | 103 | 533 | 103 | 529 | 6 |

| **Cells** | **Cells having BRCA1 foci(%)** | | **Cells having H2ax foci** | | **Colocalised foci** | | **Non colocalised** |
| --- | --- | --- | --- | --- | --- | --- | --- |
| **MDA-MB-231** | **1-5 foci** | **More foci(Number of foci)** | **1-5 foci** | **More foci(Number of foci)** | **1-5 foci** | **More foci(Number of foci)** |  |
| 1 | 3 |  | 3 |  | 3 |  |  |
| 2 | 0 |  | 0 |  | 0 |  |  |
| 3 | 0 |  | 0 |  | 0 |  |  |
| 4 | 0 |  | 0 |  | 0 |  |  |
| 5 | 3 |  | 3 |  | 3 |  |  |
| 6 |  | 10 |  | 10 |  | 10 |  |
| 7 | 3 |  | 3 |  | 3 |  |  |
| 8 |  | 11 |  | 11 |  | 11 |  |
| 9 |  | 6 |  | 6 |  | 6 |  |
| 10 |  | 6 |  | 6 |  | 6 |  |
| 11 | 1 |  | 1 |  | 1 |  |  |
| 12 | 3 |  | 3 |  | 3 |  |  |
| 13 |  |  |  |  |  |  |  |
| 14 | 1 |  | 1 |  | 1 |  |  |
| 15 | 4 |  | 4 |  | 4 |  |  |
| 16 | 5 |  | 5 |  | 5 |  |  |
| 17 | 0 |  | 0 |  | 0 |  |  |
| 18 | 4 |  | 4 |  | 4 |  |  |
| 19 | 3 |  | 3 |  | 3 |  |  |
| 20 | 1 |  | 1 |  | 1 |  |  |
| 21 | 1 |  | 0 |  |  |  | 1 |
| 22 | 5 |  | 5 |  | 5 |  |  |
| 23 | 5 |  |  | 7 |  | 7 |  |
| 24 | 4 |  |  | 6 |  | 4 | 2 |
| 25 | 1 |  | 1 |  | 1 |  |  |
| 26 | 0 |  | 0 |  | 0 |  |  |
| 27 | 1 |  | 1 |  | 1 |  |  |
| 28 |  | 6 |  | 6 |  | 6 |  |
| 29 | 3 |  | 5 |  | 3 |  | 2 |
| 30 | 4 |  | 5 |  | 4 |  | 1 |
| 31 |  | 7 |  | 7 |  | 7 |  |
| 32 | 5 |  | 5 |  | 5 |  |  |
| 33 |  | 9 |  | 9 |  | 9 |  |
| 34 |  | 7 |  | 7 |  | 7 |  |
| 35 | 1 |  | 2 |  | 1 |  | 1 |
| 36 | 2 |  | 2 |  | 2 |  |  |
| 37 | 1 |  | 1 |  | 1 |  |  |
| 38 | 1 |  | 1 |  | 1 |  |  |
| 39 |  | 7 |  | 7 |  | 7 |  |
| 40 | 2 |  | 2 |  | 2 |  |  |
| 41 | 4 |  | 4 |  | 4 |  |  |
| 42 | 2 |  | 2 |  | 2 |  |  |
| 43 | 3 |  | 3 |  | 3 |  |  |
| 44 | 2 |  | 2 |  | 2 |  |  |
| 45 |  | 10 |  | 10 |  | 10 |  |
| 46 |  | 11 |  | 11 |  | 11 |  |
| 47 | 3 |  | 3 |  | 3 |  |  |
| 48 |  | 13 |  | 13 |  | 13 |  |
| 49 |  | 8 |  | 8 |  | 8 |  |
| 50 |  | 8 |  | 8 |  | 8 |  |
| 51 | 4 |  | 3 |  | 3 |  |  |
| 52 | 3 |  | 3 |  | 3 |  |  |
| 53 | 1 |  | 1 |  | 1 |  |  |
| 54 | 3 |  | 3 |  | 3 |  |  |
| 55 | 4 |  | 4 |  | 4 |  |  |
| 56 | 2 |  | 2 |  | 2 |  |  |
| 57 | 1 |  | 1 |  | 1 |  |  |
| 58 | 4 |  | 4 |  | 4 |  |  |
| 59 | 4 |  | 4 |  | 4 |  |  |
| 60 |  | 10 |  | 10 |  | 10 |  |
| 61 |  | 0 |  | 0 |  | 0 |  |
| 62 | 2 |  | 2 |  | 2 |  |  |
| 63 | 5 |  | 0 | 6 | 5 | 1 |  |
| 64 |  | 6 |  | 6 |  | 6 |  |
| 65 | 4 |  | 4 |  | 4 |  |  |
| 66 | 3 |  | 3 |  | 3 |  |  |
| 67 | 2 |  | 2 |  | 2 |  |  |
| 68 | 3 |  | 3 |  | 3 |  |  |
| 69 | 4 |  | 4 |  | 4 |  |  |
| 70 | 2 |  | 2 |  | 2 |  |  |
| 71 | 0 |  | 0 |  | 0 |  |  |
| 72 | 0 |  | 0 |  | 0 |  |  |
| 73 | 4 |  | 4 |  | 4 |  |  |
| 74 |  | 10 |  | 10 |  | 10 |  |
| 75 | 3 |  | 3 |  | 3 |  |  |
| 76 | 3 |  | 3 |  | 3 |  |  |
| 77 | 0 |  | 0 |  | 0 |  |  |
| 78 | 0 |  | 0 |  | 0 |  |  |
| 79 |  | 6 |  | 6 |  | 6 |  |
| 80 |  | 8 |  | 8 |  | 8 |  |
| 81 | 1 |  | 1 |  | 1 |  |  |
| 82 | 1 |  | 1 |  | 1 |  |  |
| 83 |  | 8 |  | 8 |  | 8 |  |
| 84 | 2 |  | 2 |  | 2 |  |  |
| 85 | 1 |  | 1 |  | 1 |  |  |
| 86 | 1 |  | 1 |  | 1 |  |  |
| 87 | 1 |  | 1 |  | 1 |  |  |
| 88 | 2 |  | 2 |  | 2 |  |  |
| 89 | 0 |  | 0 |  | 0 |  |  |
| 90 | 2 |  | 2 |  | 2 |  |  |
| 91 |  | 10 |  | 10 |  | 10 |  |
| 92 | 2 |  | 2 |  | 2 |  |  |
| 93 | 1 |  | 1 |  | 1 |  |  |
| 94 | 3 |  | 3 |  | 3 |  |  |
| 95 | 1 |  | 1 |  | 1 |  |  |
| 96 | 3 |  | 3 |  | 3 |  |  |
| 97 | 2 |  | 2 |  | 2 |  |  |
| 98 | 1 |  | 1 |  | 1 |  |  |
| 99 |  | 7 |  | 7 |  | 7 |  |
| 100 | 3 |  | 3 |  | 3 |  |  |
| 101 | 1 |  | 1 |  | 1 |  |  |
| 102 |  | 6 |  | 6 |  | 6 |  |
| **Total** | 170 | 190 | 158 | 209 | 159 | 202 | 7 |

| **Cells** | **Cells having BRCA1 foci(%)** | | **Cells having H2ax foci** | | **Colocalised foci** | | **Non colocalised** |
| --- | --- | --- | --- | --- | --- | --- | --- |
| **MCF-7** | **1-5 foci** | **More foci(Number of foci)** | **1-5 foci** | **More foci(Number of foci)** | **1-5 foci** | **More foci(Number of foci)** |  |
| 1 | 1 |  | 1 |  | 1 |  |  |
| 2 |  | 12 |  | 12 |  | 12 |  |
| 3 |  | 9 |  | 9 |  | 9 |  |
| 4 |  | 8 |  | 8 |  | 8 |  |
| 5 | 3 |  | 3 |  | 3 |  |  |
| 6 |  | 7 |  | 7 |  | 7 |  |
| 7 | 2 |  | 2 |  | 2 |  |  |
| 8 |  | 8 |  | 8 |  | 8 |  |
| 9 |  | 9 |  | 9 |  | 9 |  |
| 10 | 5 | 0 |  | 5 |  | 5 |  |
| 11 | 3 |  | 3 |  | 3 |  |  |
| 12 | 4 |  | 4 |  | 4 |  |  |
| 13 | 2 |  | 2 |  | 2 |  |  |
| 14 | 2 |  | 2 |  | 2 |  |  |
| 15 | 3 |  | 3 |  | 3 |  |  |
| 16 | 4 |  | 4 |  | 4 |  |  |
| 17 | 5 |  | 5 |  | 5 |  |  |
| 18 | 3 |  | 3 |  | 3 |  |  |
| 19 | 0 |  | 0 |  | 0 |  |  |
| 20 | 1 |  | 1 |  | 1 |  |  |
| 21 | 3 |  | 3 |  | 3 |  |  |
| 22 | 0 |  | 0 |  | 0 |  |  |
| 23 |  | 7 |  | 7 |  | 7 |  |
| 24 |  | 6 |  | 6 |  | 6 |  |
| 25 | 1 |  | 1 |  | 1 |  |  |
| 26 | 1 |  | 1 |  | 1 |  |  |
| 27 | 1 |  | 1 |  | 1 |  |  |
| 28 |  | 6 |  | 6 |  | 6 |  |
| 29 |  | 6 |  | 6 |  | 6 |  |
| 30 | 1 |  | 1 |  | 1 |  |  |
| 31 | 1 |  | 1 |  | 1 |  |  |
| 32 |  | 6 |  | 6 |  | 6 |  |
| 33 |  | 8 |  | 8 |  | 8 |  |
| 34 |  | 11 |  | 11 |  | 11 |  |
| 35 |  | 6 |  | 6 |  | 6 |  |
| 36 |  | 6 |  | 6 |  | 6 |  |
| 37 |  | 7 |  | 7 |  | 7 |  |
| 38 | 4 |  | 4 |  | 4 |  |  |
| 39 | 2 |  | 2 |  | 2 |  |  |
| 40 |  | 10 |  | 10 |  | 10 |  |
| 41 |  | 11 |  | 11 |  | 11 |  |
| 42 |  | 12 |  | 12 |  | 12 |  |
| 43 | 5 |  | 5 |  | 5 |  |  |
| 44 |  | 11 |  | 11 |  | 11 |  |
| 45 | 3 |  | 3 |  | 3 |  |  |
| 46 | 4 |  | 4 |  | 4 |  |  |
| 47 |  | 10 |  | 10 |  | 10 |  |
| 48 | 1 |  | 1 |  | 1 |  |  |
| 49 | 5 |  | 5 |  | 5 |  |  |
| 50 | 5 |  | 5 |  | 5 |  |  |
| 51 |  | 7 |  | 7 |  | 7 |  |
| 52 |  | 9 |  | 9 |  | 9 |  |
| 53 |  | 6 |  | 6 |  | 6 |  |
| 54 | 4 |  | 4 |  | 4 |  |  |
| 55 |  | 8 |  | 8 |  | 8 |  |
| 56 | 0 |  | 0 |  |  | 0 |  |
| 57 |  | 8 |  | 8 |  | 8 |  |
| 58 |  | 7 |  | 7 |  | 7 |  |
| 59 |  | 9 |  | 9 |  | 9 |  |
| 60 |  | 10 |  | 10 |  | 10 |  |
| 61 |  | 15 |  | 15 | 0 | 15 |  |
| 62 | 5 |  | 5 |  | 5 |  |  |
| 63 | 2 |  | 2 |  | 2 |  |  |
| 64 | 3 |  | 3 |  | 3 |  |  |
| 65 |  | 9 |  | 9 |  | 9 |  |
| 66 | 3 |  | 3 |  | 3 |  |  |
| 67 | 4 |  | 4 |  | 4 |  |  |
| 68 |  | 6 |  | 6 |  | 6 |  |
| 69 |  | 10 |  | 10 |  | 10 |  |
| 70 | 0 |  | 0 |  | 0 |  |  |
| 71 | 3 |  | 3 |  | 3 |  |  |
| 72 |  | 11 |  | 11 |  | 11 |  |
| 73 |  | 9 |  | 9 |  | 9 |  |
| 74 |  | 8 |  | 8 |  | 8 |  |
| 75 |  | 8 |  | 8 |  | 8 |  |
| 76 |  | 7 |  | 7 |  | 7 |  |
| 77 | 5 |  | 5 |  | 5 |  |  |
| 78 |  | 10 |  | 10 |  | 10 |  |
| 79 |  | 9 |  | 9 |  | 9 |  |
| 80 |  | 8 |  | 8 |  | 8 |  |
| 81 |  | 9 |  | 9 |  | 9 |  |
| 82 | 3 |  | 3 |  | 3 |  |  |
| 83 | 4 |  | 4 |  | 4 |  |  |
| 84 |  | 6 |  | 6 |  | 6 |  |
| 85 | 0 | 10 |  | 10 |  | 10 |  |
| 86 |  | 7 |  | 7 |  | 7 |  |
| 87 | 5 |  | 5 |  | 5 |  |  |
| 88 | 1 |  | 1 |  | 1 |  |  |
| 89 | 5 |  | 5 |  | 5 |  |  |
| 90 | 3 |  | 3 |  | 3 |  |  |
| 91 | 1 |  | 1 |  | 1 |  |  |
| 92 | 1 |  | 1 |  | 1 |  |  |
| 93 | 2 |  | 2 |  | 2 |  |  |
| 94 |  | 10 |  | 10 |  | 10 |  |
| 95 |  | 9 |  | 9 |  | 9 |  |
| 96 | 4 |  | 4 |  | 4 |  |  |
| 97 |  | 9 |  | 9 |  | 9 |  |
| 98 | 4 |  | 4 |  | 4 |  |  |
| 99 | 5 |  | 5 |  | 5 |  |  |
| 100 |  | 12 |  | 12 |  | 12 |  |
| 101 |  | 9 |  | 9 |  | 9 |  |
| **Total** | 142 | 431 | 137 | 436 | 137 | 436 |  |
