# Supplementary material for: BRCA1 interactors, RAD50 and BRIP1, as prognostic markers for triple-negative breast cancer severity
Source: Front Genet. 2023 Feb 16;14:1035052. doi: 10.3389/fgene.2023.1035052 (PMC9978165; doi:10.3389/fgene.2023.1035052)
Supplement: Supplementary file 6 [file DataSheet1.DOCX]

**Figure1. Differential expression of TP53 and BRCA1 within TNBC cells. A and B. *BRCA1 and TP53 expression changes with TNBC condition:* The bar graph represents the expression studies done with** c-DNA (500 ng of RNA) from different breast cancer lines, TNBC cell line MDA-MB-468 and MDA-MB-231and luminal type MCF-7. The expression was quantified from the technical triplicates of the RT qPCR data. **C.** ***Association of BRCA1 and TP53 shows up in TNBC severe condition only:*** Association between BRCA1 and TP53 in different BC tissues conditions obtained from TCGA data are analyzed by correlogram. Moving towards darker blue colour indicates an increase in positive correlation, whereas towards darker red indicates an increase in negative correlation. The white colour indicates no correlation. **D and E**. ***TP53 and its functioning are compromised in some TNBC conditions only:*** TP53 expression, as well as its phosphorylation, was detected by western blot analysis of proteins extracted from the above mentioned cells, using corresponding (TP53 and phosphor-TP53) antibodies. ß-actin is given as the loading control. **F. *Quantification of TP53 and pTP53 expression in breast cancer cells:*** The intensity of the TP53 and its phosphorylated bands from western blots were quantified using Image J software. The values of band intensities were normalized with the corresponding ß-actin to normalize the protein loading. **G.** ***Functioning of BRCA1 is also compromised in MDA-MB-231 condition***: Functioning of BRCA1 molecule is visualized by the formation of BRCA1 foci in all the different cell lines in the Immunofluorescence assay.**H. *Quantification of BRCA1-foci in TNBC cells:*** BRCA1-foci formation is scored in each cell and plotted as a bar graph. The X-axis represents the different cell line and Y-axis represents the total number of BRCA1 foci formed in 200 cells. All the experiments are done in biological triplicates. Data were analysed by one-way ANOVA, Pearson correlation test and unpaired T-test. (SEM is shown as error bars. **P*< 0.05, ** P < 0.005 and ^#^ *P*<0.0001)

**Figure 2. The integrity of the genetic material differs between TNBC lines.  A**. ***MDA-MB-231 cells have a significantly less G2/M population:*** Cell cycle analysis for different breast cancer cell lines, MDA-MB-468, MDA-MB-231 and MCF-7 , was performed in a flow-cytometer. The histogram plot represents the distribution of 5000 cells into different phases of the cell cycle. **B.** ***Quantification of the cell population at different stages:*** The population of cells in different phases of the cell cycle were analyzed and plotted in a bar graph using In-cyte software of GUAVA Easycyte. **C. *Heterogeneous arrangement of the genetic material in MDA-MB-231 cells suggests DNA damage*: Representative** cell images (100X magnification) of the above mentioned lines stained with DAPI show the homogeneous and heterogeneous arrangement of the genetic material. **D. *Quantification of the increase in heterogeneity in the DNA of MDA-MB-231 cells:*** The bar graph represents the qualitative analysis of around 200 cells with heterogeneous (blue patches) and homogeneous (evenly spread blue) DNA. **E.** ***Increase in tail formation in MDA-MB-231 confirms more DNA damage:*** Representative image of cells having tails of fragmented DNA (comet) shows the extent of DNA damage in different cell types. The cells with the elongated tails are marked with broken red line arrows in the figure. **F. *Quantification of the number of cells having tail DNA:*** The cells having comet (in all the fields captured) are plotted in the bar graph. A total of 150 cells were counted for each cell line. **G**. ***The fragmented DNA in MDA-MB-231 cells moved the fastest through Agarose Gel electrophoresis:*** The figure represents the movement of DNA through Agarose gel ran at low voltage for a longer time. The migrated distance from the starting point 0, to the mid of the DNA band marked as 1, 2 and 3 were measured. The distance traversed by MCF-7, MDA-MB-468 and MDA-MB-231 was 24.45 mm, 25.40 mm and 29.90 mm respectively. All the experiments were performed in biological triplicates. Data were analyzed by one-way ANOVA. (SEM is shown as an error bar. **P*< 0.05, ** P < 0.005, *** P < 0.0005 and ^#^ *P*<0.0001)

**Figure 3. DNA damage sensing abilities changes between different TNBC lines. A. *Rad50 expression is compromised in MDA-MB-231 cells:* RAD50 expression** was detected by western blot analysis of proteins extracted from breast cancer line, TNBC subtypes MDA-MB-468 and MDA-MB-231, and luminal type cell line MCF-7 using antibodies directed against Rad50 protein. ß-actin is considered as the loading control. **B.** ***Quantification of RAD50 protein expression in all the cell types:*** The intensity of the RAD50 bands from the western analysis were quantified using Image J software. The values of band intensities were normalized with corresponding intensities of ß -actin. ***C.*** ***MDA-MB -231 cells showed compromised expression of RAD50 transcript also:*** **Expression studies were done with** c-DNA (500 ng of RNA) from the above mentioned cell lines. The RAD50 expression was quantified by Real-time PCR from the technical triplicates and represented in the bar graph. ∆∆Ct value was calculated from the C_t_ value of GAPDH and No template control (NTC). **D. *Formation of less H2AX foci in MDA-MB-231:*** Representative fields of immunofluorescence assay showing gamma-H2AX and BRCA1 foci. The red dots in the first panel indicates the gamma-H2AX foci, Green dots in the second panel indicates the BRCA1 foci, **yellow in the third panel indicated the co-localized foci and b**lue in the fourth panel shows the co-localization of H2AX-BRCA1 foci with the nucleus (DAPI) **E.** ***The colocalization of BRCA1 with H2AX is mildly compromised in MDA-MB-231:*** The percentage of cells with no foci and co-localized foci was analyzed by counting 200 cells and represented in the bar graph. ***F.*** ***H2AX- BRCA1 foci as the marker for DNA damage sensing, are significantly compromised in MDA-MB-231:*** The foci formation is analysed manually by scoring the red and green dots in 200 cells. MDA-MB-231 with less H2AX BRCA1 foci confirms the defect in damage sensing. All the experiments in this figure were performed in biological triplicates. Data were analysed by one-way and two-way ANOVA. (SEM is shown as an error bar. **P*< 0.05, ** P < 0.005 and *** P < 0.0005)

**Figure 4. Up regulation of the repair pathway due to accumulated damage:** **A and B. *Over expressed HR and NHEJ proteins in MDA-MB-231 cells.*** HR marker BRIP1 and NHEJ marker KU70 were detected by western blot analysis of proteins extracted from breast cancer lines, TNBC sub types MDA-MB-468 and MDA-MB-231, and luminal type cell line MCF-7 using corresponding antibodies. ß-actin is given as the loading control. **C. *Quantification of repair proteins in all the cell types***. The intensity of the BRIP1 and KU70 bands from the western analysis were quantified using Image J software. The values of band intensities were normalized with corresponding intensities of ß-actin. **D. *The transcript level of BRIP1 is similar in TNBC cells:* Expression studies were done with** c DNA (500 ng of RNA) from the above lines. The bar graph represents the similar expression of BRIP1 in both the TNBC lines which were quantified by Real-time qPCR from the technical triplicates and represented in the bar graph. **E*. KU70 is over expressed in MDA-MB-231 cells compared to other TNBC lines.*** The functional analysis of KU70 was done with the extracted RNA from the above cell lines. The bar graph represents the expression of KU70 significantly more in case of MDA-MB-231. The analysis was done with technical triplicates. ∆∆Ct value was calculated from the Ct value of GAPDH, the housekeeping gene and no template control (NTC), the non-specific background. **F.** ***Robust*** ***DNA damage-recovery in MDA-MB-231 due to the presence of over expressed Ku70.*** The cells were treated with 0.035% MMS for 15 minutes and released in MMS free media. Cells were harvested at 0h and 24h and the movement of DNA contents are visualized through Agarose Gel electrophoresis. The figure represents the movement of DNA through Agarose gel ran at 25 voltage for 22 hours. **G. *Percentage of BRCA1-H2AX foci formation in MMS treated cells visualized by Immunofluorescence assay.*** The foci formation is analyzed manually by scoring the green (BRCA1) and red (H2AX) dots in MMS untreated cells and 0 h/24h post MMS treatment cells(recovered). HR compromised cells MDA-MB-231 cells showing similar repair activity as HR efficient cells (MDA-MB-468) due to over expressed NHEJ repair, justifying the robust repair. The X-axis represents the different cells and Y-axis represents the percentage of BRCA1- H2AX foci. **H. *Association of BRCA1/ TP53 with BRIP1 shows up only in TNBC severe condition.***Association between BRCA1, TP53 and BRIP1 in different tissues condition like the normal solid sample (N=119), ER/PR+ve (N=314), TNBC (N=95) and TNBC severe (N=9) is analyzed by UCSC-Xena (TCGA) and represented in correlogram. Moving towards darker blue colour indicates an increase in positive correlation, whereas towards darker red indicates an increase in negative correlation. The White colour indicates no correlation. All the experiments in this figure were performed in biological triplicates. Data were analyzed by one-way ANOVA and Pearson correlation test. (SEM is shown as error bar. **P*< 0.05 and ** P < 0.005)

**Figure 5: Severity is highly dependent on repair pathways. A. K*i67 is over expressed in MDA-MB-231 cells compared to other breast cancer sub types.***The proliferation of different breast cancer lines, TNBC sub types MDA-MB-468 and MDA-MB-231, and luminal type cell line MCF-7 were monitored by measuring the Ki67 marker using Immunophenotyping assay and represented by histogram plots (Black line indicates the unstained population and the blue line indicates the Ki67 stained population). **B. *Quantification of the Ki67 population in different cell types.***The bar graph represents the percentages of cells having Ki67 expression which was measured by gating the Ki67 population using GUAVA in-cyte software. **C*. The angiogenic/metastatic factor in Breast Cancer, BACH1 over expresses in MDA-MB-231 cells.*** The protein expression of metastasis marker BACH1 was detected by western blot analysis of proteins extracted from the above mentioned cells, using the corresponding antibody. ß-actin is given as the loading control. **D**. ***Quantification of BACH1 protein using ImageJ software.***  The bar graph represents the intensity of BACH1 bands from western analysis which was quantified using Image J software. The values of band intensities were normalized with corresponding intensities of ß-actin. **E.** ***MDA-MB-231 cells showed over expression of BACH1 transcript also:*** **Expression studies were done with** c-DNA (500 ng of RNA) from the above mentioned cell lines. The BACH1 expression was quantified by RT- PCR from the technical triplicates and represented in the bar graph. ∆∆Ct value was calculated from the C_t_ value of GAPDH and no template control (NTC). **F.**  ***MDA-MB-231 has more cancer stem cell population:*** The cancer stem cell population was analysed by Immunophenotyping assay. The dot plots represent the CD44+ve/CD24-ve stained (upper panel) and unstained (lower panel) population in different cell types. **G**. ***Quantification of the higher population of cancer stem cells in MDA-MB-231:*** The bar graph represents the percentages of cells having CD44+ve/CD24-ve expression which was measured by gating the CD44+ve/CD24-ve population using GUAVA incyte software. All the experiments were performed in biological triplicates. Data were analysed by one-way ANOVA. (SEM is shown as error bar. **P*< 0.05 and ** P < 0.005)
